# A machine learning framework to classify musculoskeletal injury risk groups in military service members

**DOI:** 10.3389/frai.2024.1420210

**Published:** 2024-06-19

**Authors:** Matthew B. Bird, Megan H. Roach, Roberts G. Nelson, Matthew S. Helton, Timothy C. Mauntel

**Affiliations:** ^1^Extremity Trauma and Amputation Center of Excellence, Defense Health Agency, Falls Church, VA, United States; ^2^Department of Clinical Investigations, Womack Army Medical Center, Fort Liberty, NC, United States; ^3^Department of Surgery, Uniformed Services University of the Health Sciences, Bethesda, MD, United States; ^4^Artificial Intelligence Integration Center, Army Futures Command, Pittsburgh, PA, United States; ^5^U.S. Army, Tripler Army Medical Center, Honolulu, HI, United States

**Keywords:** survival, Cox proportional hazard regression, decision trees, screening, secondary data

## Abstract

**Background:**

Musculoskeletal injuries (MSKIs) are endemic in military populations. Thus, it is essential to identify and mitigate MSKI risks. Time-to-event machine learning models utilizing self-reported questionnaires or existing data (e.g., electronic health records) may aid in creating efficient risk screening tools.

**Methods:**

A total of 4,222 U.S. Army Service members completed a self-report MSKI risk screen as part of their unit's standard in-processing. Additionally, participants' MSKI and demographic data were abstracted from electronic health record data. Survival machine learning models (Cox proportional hazard regression (COX), COX with splines, conditional inference trees, and random forest) were deployed to develop a predictive model on the training data (75%; *n* = 2,963) for MSKI risk over varying time horizons (30, 90, 180, and 365 days) and were evaluated on the testing data (25%; *n* = 987). Probability of predicted risk (0.00–1.00) from the final model stratified Service members into quartiles based on MSKI risk.

**Results:**

The COX model demonstrated the best model performance over the time horizons. The time-dependent area under the curve ranged from 0.73 to 0.70 at 30 and 180 days. The index prediction accuracy (IPA) was 12% better at 180 days than the IPA of the null model (0 variables). Within the COX model, “other” race, more self-reported pain items during the movement screens, female gender, and prior MSKI demonstrated the largest hazard ratios. When predicted probability was binned into quartiles, at 180 days, the highest risk bin had an MSKI incidence rate of 2,130.82 ± 171.15 per 1,000 person-years and incidence rate ratio of 4.74 (95% confidence interval: 3.44, 6.54) compared to the lowest risk bin.

**Conclusion:**

Self-reported questionnaires and existing data can be used to create a machine learning algorithm to identify Service members' MSKI risk profiles. Further research should develop more granular Service member-specific MSKI screening tools and create MSKI risk mitigation strategies based on these screenings.

## Introduction

Musculoskeletal injuries (MSKIs) impose a burden on the United States (U.S.) Military Healthcare System (MHS). MSKIs account for more than 2 million medical encounters annually across the U.S. military (Teyhen et al., 2014) and cost the MHS more than $500 million dollars per year (Teyhen et al., 2018). More importantly, MSKIs limit military Service members' abilities to train and perform required work duties (Ruscio et al., 2010; Teyhen et al., 2018). Despite the significant negative impact MSKIs have on operational readiness and the financial burden on the MHS, there are few actionable and deployable MSKI risk classification models that can help inform clinical practice to reduce MSKI risks among Service members (Rhon et al., 2022b).

Multi-factorial MSKI risk modeling has been the focus of MSKI risk research for the past decade (Bahr, 2016; Bittencourt et al., 2016). Commonly, previous MSKI risk modeling research has discretized variables before modeling [e.g., body mass index (BMI): underweight, healthy, overweight, and obese] to simplify a model's clinical interpretability (Teyhen et al., 2020); however, discretization is not recommended to optimize model performance (Carey et al., 2018). MSKI risk assessment models that are overly simplistic, demonstrate poor model performance, are not yet validated in a new cohort, or are too complex for interpretation, which may misguide clinicians (Bullock et al., 2021, 2022). However, validated machine learning models that provide clinically relevant results are attainable with the correct tools and proper framework.

Machine learning models, built-in frameworks designed for modularity (e.g., flexibility in the prediction model and/or variables included in the model) and scalability (e.g., handling large amounts of data), may have the greatest opportunity for deployment in clinical or operational settings. In addition, machine learning models that are flexible (e.g., random forests) may further allow for modularity and account for non-linearity in the data when compared to more traditional statistical approaches (e.g., regressions). Machine learning models are typically built across a specific time horizon (e.g., within 1 year) and do not account for varying time horizons (i.e., exposure to time), which can limit their utility in clinical settings (Van Eetvelde et al., 2021). Thus, other models must be employed to address the complexity of MSKI risk data.

Time-to-event or survival models estimate the probability of an event occurring before a specified time horizon (Nielsen et al., 2019a). Unlike binary classification models (e.g., logistic regression), which make predictions at a one-time horizon, survival models predict MSKI risk across a continuous range of time horizons accounting for censored individuals. The most common of these survival models is the Cox proportional hazard model. The major limitation of the Cox model is that it makes a strong assumption that the hazard functions between individuals are proportional (Andersen, 2022). Thus, to account for censoring and to utilize flexible survival models (e.g., decision trees), it is necessary to look to other fields deploying survival machine learning techniques for potential applications for MSKI risk stratification (Wang et al., 2019). These existing techniques deployed in MSKI risk stratification may better enable clinicians to estimate survival probability outcomes (i.e., time-to-MSKI) in a methodologically valid and flexible framework.

The purpose of this study was to determine if self-reported questionnaires and existing data sources (e.g., demographics) from military Service members could be used to create a modular machine learning model for assessing MSKI risk. The use of existing self-report data is essential to make the models scalable to a large number of U.S. military Service members [~1.3 million active-duty Service members (U.S. Department of Defense, 2022)]. Second, we will assess varying statistical modeling approaches to provide guidance for appropriate tools to classify MSKI risk in a machine learning framework. We hypothesize that a high-throughput self-report MSKI risk screen, combined with existing data sources, will provide the data necessary to build a machine learning model with acceptable model performance in identifying Service members' MSKI risk levels.

## Materials and methods

We conducted a retrospective review of existing self-report MSKI screen data and electronic medical records to develop MSKI risk screening models. A total of 4,222 Service members completed a self-report MSKI risk screen as part of their standard in-processing to a U.S. Army Airborne Division. Complete datasets were accessible for 3,950 Service members (female gender: =12.2%, age: 24.1 ± 5.6 years, height: 175.4 ± 8.8 cm, mass: 78.71 ± 12.0 kg) and were used for analyses. This retrospective cohort study was deemed exempt from the Institutional Review Board review by the Womack Army Medical Center Human Research Protections Program Office.

### Data types and reduction

Military Service members in-processing to a U.S. Army Airborne Division completed a self-report MSKI risk screen between December 2020 and March 2022. The self-reported questionnaire consisted of general health, physical fitness, and demographic-type questions (Table 1) (Roach et al., 2023). Additionally, Service members self-reported if they experienced pain during three movement screens: (1) shoulder clearing (bilateral), (2) spinal extension clearing, and (3) squat-jump-land. Pain with either shoulder clearing screen (i.e., left or right) was documented as one, regardless of whether the pain was experienced unilaterally or bilaterally. The three movement screen pain items were summed for a total movement screen pain score (score range = 0–3) for each Service member. The movement screen assessment methods have been previously described (Roach et al., 2023).

**Table 1:** Model features.

**Variable**	**Data structure**	**Data source**	**Data**
Mass	Continuous	Self-reported	Kilograms
Height	Continuous	Self-reported	Centimeters
Sleep	Continuous	Self-reported	Hours
Prior MSKI	Factor	Self-reported	Yes, no^*^
Prior surgery	Factor	Self-reported	Yes, no^*^
MSKI profile	Factor	Self-reported	Yes, no^*^
Stress fracture	Factor	Self-reported	Yes, no^*^
Last ACFT pain	Factor	Self-reported	Yes, no^*^
Last ACFT failure	Factor	Self-reported	Yes, no^*^
Nicotine use	Factor	Self-reported	Yes, no^*^
Paygrade	Factor	Self-reported	E1-E4, E5-E10, Officer^*^
Movement screen pain	Factor	Self-reported	0^*^, 1, 2, or 3
Age	Continuous	DEERS	Years
Marital status	Factor	DEERS	Married, single^*^
DoD occupation code	Factor	DEERS	Combat arms^*^, combat support, combat services and support
Race	Factor	DEERS	White^*^, Asian or Pacific Islander, Black, American Indian or Alaskan native, other, unknown
Gender	Factor	DEERS	Male^*^, female

Variable: mass, converted from pounds to kilograms; height, converted from inches to centimeters; sleep, average duration of sleep; prior MSKI, any MSKI within the previous year; prior surgery, any prior surgery within 2 years requiring physical therapy; MSKI profile, limited duty profile status within 1 year that resulted in 3 or more days of missed/altered activity; stress fracture, any stress fracture over a Service member's lifetime; last ACFT pain, pain during the last attempted ACFT; last ACFT failure, failing score on any event in the last attempted ACFT; nicotine use, current or previous nicotine use; movement screen pain, pain during the included movement screens; DoD occupation code, occupation index codes categorized into three groups.

Data source: self-reported, self-reported questionnaire administered at the start of the Service member's surveillance period start date. DEERS, data extracted from M2 closest to the Service member's surveillance period start date. Referent categories are labeled with an (^*^) under the Data column.

Occupation codes, age, race, gender, and marital status were collected via the Defense Enrollment Eligibility Reporting System (DEERS) within the MHS Management Analysis and Reporting Tool (MHS MART [M2]) closest to the Service member's in-processing date (Table 1). The occupation index codes were categorized into three groups: (1) combat arms (e.g., infantry and field artillery); (2) combat support (e.g., artillery repair and counterintelligence); and (3) combat service support (e.g., medical, instructors, and transportation) (Teyhen et al., 2018).

The MSKI data [International Classification of Diseases - Tenth Revision (ICD-10 Codes)] were collected via the Comprehensive Ambulatory Provider Encounter Record (CAPER) within M2 from the time of the Service member's in-processing screening date up to 1 year post-screening. The CAPER provides direct care outpatient encounter records. Thus, any MSKI treated and diagnosed by an MHS healthcare provider is documented in the CAPER. MSKI encounters were labeled via an MSKI classification matrix (Hando et al., 2023) using the ICD-10 Codes extracted from CAPER. Days until MSKI were calculated as the date of the first MSKI encounter minus the in-processing date. For analysis, subjects were right-censored if they did not sustain an MSKI (noMSKI) within 1 year from their in-processing date, and no duplicate Service members were present in the analysis.

### Data analysis: model performance measurements

The area under the curve (AUC) is defined as the area under the receiver operating characteristic (ROC) curve, which quantifies the discriminative power of a variable or multivariable model to correctly classify outcome occurrences (e.g., MSKI) across all probability thresholds. Because MSKI occurrence changes over time, the time-dependent AUC (t-AUC) is used to evaluate model discrimination as a function of time (Heagerty et al., 2000). The t-AUC values range from 0.50 to 1, with 1 being perfect discrimination and 0.5 being a random guess.

Calibration is a measurement of the agreement between the predicted probability of the model and the actual risk. A perfectly calibrated model will have prediction probabilities that exactly match the actual risk outcomes in the data. When plotting actual risk (*y*-axis) against predicted risk (*x*-axis), perfect calibration is marked by the line *y* = *x*. Any deviation from this line indicates an over- or under-optimistic model. In survival data with censoring, the actual risk is not directly observed, so we estimate it using the Kaplan–Meier estimate (Austin et al., 2020).

The Brier score measures both model discrimination and calibration in one metric. In the absence of censoring, the Brier score is the mean-squared error for binary outcomes. In the presence of right-censoring, the Brier score is weighted with an inverse probability of censoring. A Brier score of 0 indicates a perfectly accurate model, a score of 1 indicates a perfectly inaccurate model, and a score of 0.25 indicates a randomly guessing model if the incidence was 50%. Because the incidence changes over time in survival models, the Brier score for a random guess will also change. To account for this change, the scaled Brier score, or index prediction accuracy (IPA), is defined as the percent improvement over random guess, where 0% is an uninformative model and 100% is a perfectly accurate model (Kattan and Gerds, 2018).

### Data analysis: survival models

The missing data were evaluated before analysis and were determined to be low (pay grade: 5.5%, occupation: 1.0%, race: 0.6%, height: 0.1%). Thus, complete cases (*n* = 3,950) were used for analyses. The data were randomly split into training (75%) and test (25%) sets with an equal proportion of Service members with MSKI in each set. The Cox proportional hazard regression (COX), COX with splines (COX-S), conditional inference trees (CTREE), and random forest (RF) were used to train multiple models on the training data set. All models accounted for right-censored data utilizing a survival function, and the models were trained and tuned individually.

COX generates multivariable survival models that allow multiple variables (continuous or categorical) to be simultaneously assessed via survival probability. Hazard ratios were calculated to provide event rates in one group compared to the other (Deo et al., 2021). Two COX models were developed; COX included all features in their raw format without transformation, while COX-S applied a restricted cubic spline basis expansion to continuous features where the number of knots on each predictor was determined by selecting the model with the lowest Akaike's information criterion (AIC) (Rutherford et al., 2015). The number of knots for each predictor was allowed to take on integer values between 1 and 5.

The CTREE algorithm generates a single binary decision tree where the split at each node is based on the *p*-value of a statistical test. In the case of survival outcomes, the splits are made using the log-rank test (Hothorn et al., 2015). The significance criterion hyperparameter for the CTREE model was selected using 10-fold cross-validation to maximize the t-AUC at 180 days. The significance criterion hyperparameter was evaluated at the following values: 0.8, 0.85, 0.9, 0.95, and 0.99.

The RF algorithm creates an ensemble of decision trees that fit the bootstraps of the data, so predictions are made based on majority voting across the ensemble (Biau and Scornet, 2016). RF is extended to survival data by approximating the cumulative hazard function in each leaf node with the Nelson–Aalen estimator and splitting nodes based on maximizing the concordance index of the tree (Ishwaran et al., 2008). The hyperparameters for the RF model were tuned by maximizing the out-of-bag concordance index over a grid search for the minimum number of observations per node (3, 5, 7, and 9) and the number of features to consider at each split (2, 4, 6, 8, and 10). The final model was trained with these chosen hyperparameters and an ensemble size of 500 trees.

After tuning model hyperparameters, all models were trained on the full training set and evaluated on the testing data. Model performances on the test set were evaluated using the t-AUC and scaled Brier score for time horizons between 30 and 365 days. The final model selection was performed by minimizing the Brier score at 180 days. Based on this criterion, the COX model was selected for risk stratification.

### Data analysis: musculoskeletal injury risk stratification

**Figure 1:**
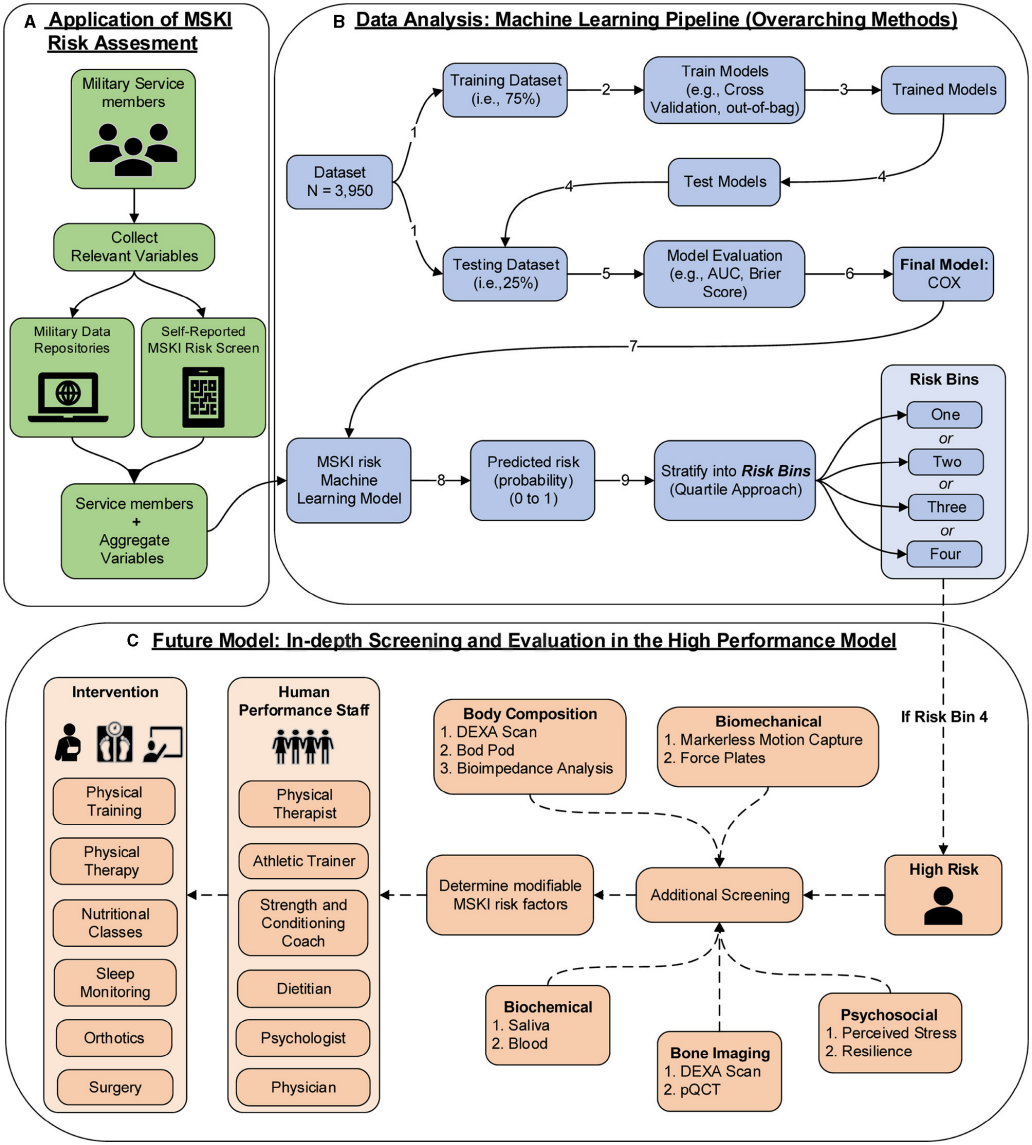
MSKI risk assessment framework in the military; **(A)** application of MSKI risk assessment (*green*): collect relevant existing data on service members; **(B)** data analysis: machine learning pipeline (*blue*): data analysis pipeline utilized for the creation of machine learning models in the manuscript. *In application*, the Service member's data would be inputted into the MSKI risk machine learning model for categorization of the risk bin; **(C)** future model: in-depth screening and evaluation in the high-performance model (*orange*): *In application*, if the service member is flagged in risk bin four, then further screening assessments are performed to determine if any modifiable risk factors can be addressed by the human performance staff for further intervention.

The COX model's calibration curve was binned by quartiles to create four equal “risk bins” to compare predicted risk to estimated risk in each bin across different time horizons (30, 90, 180, and 365 days). Calibration was assessed qualitatively by comparing the mean predicted risk in each group with their Kaplan–Meier estimate. Incidence rates (IRs) and incidence rate ratios (IRRs) were used to compare MSKI incidences across risk bins and time horizons (risk bin 1 served as the reference). Comparisons were made across the risk bins using Fisher's exact test for categorical features and the one-way ANOVA for continuous features. Additional *post-hoc* comparisons were performed by comparing risk bin 1 to risk bin 2, risk bin 3, and risk bin 4 with a Holm–Bonferroni adjustment on the *p*-values. See Figure 1 (data analysis: machine learning pipeline) for our data analysis framework. R version 4.2 was used for all statistical analysis, namely packages caret, riskRegression, tidymodels, survival, party, ranger, dplyr, readr, ggfortify, and gridExtra (R Core Team, 2019). R scripts are provided in Supplementary material.

## Results

**Figure 2:**
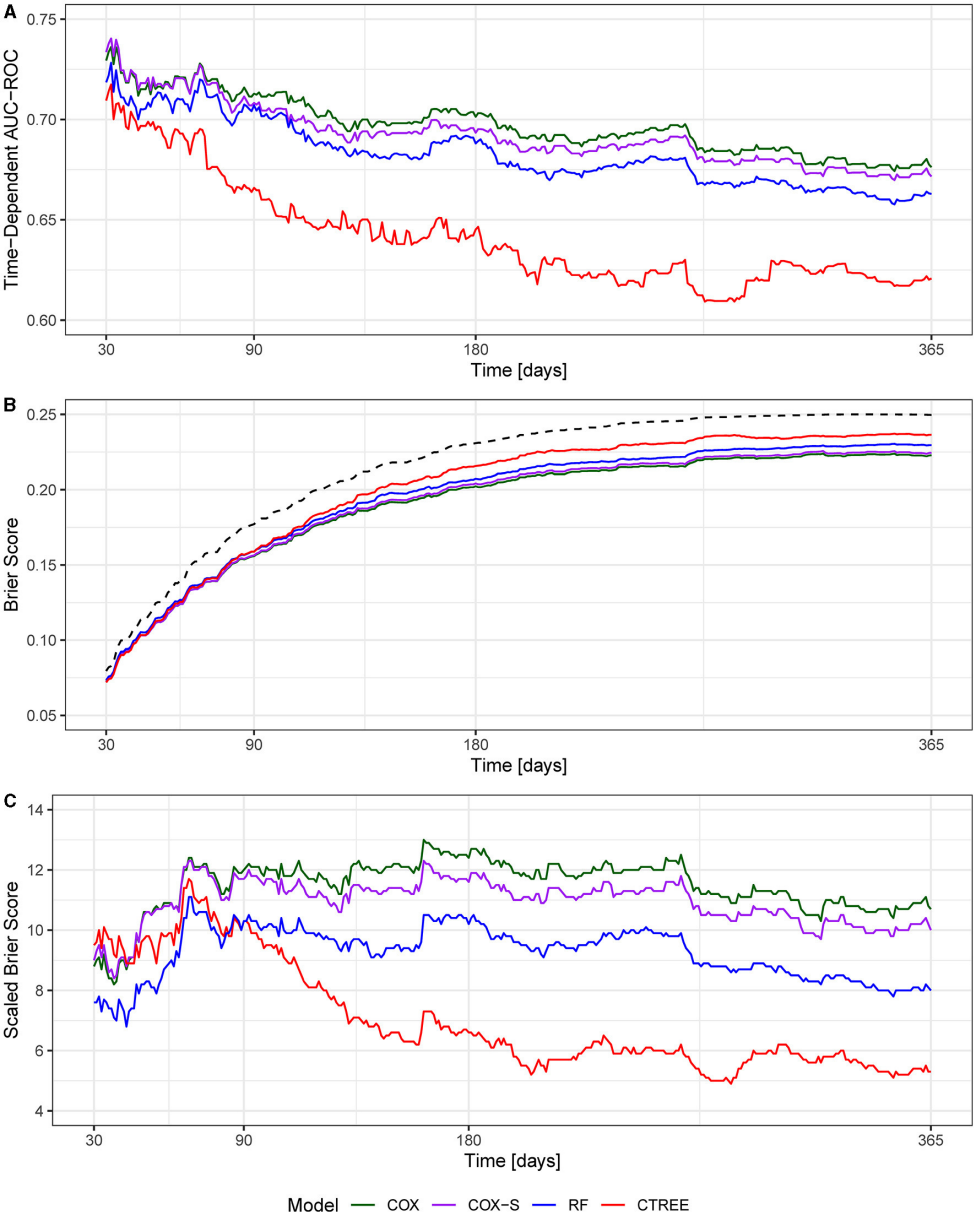
**(A)** Time-dependent area under the curve receiver operator characteristic curve (t-AUC); **(B)** absolute Brier score, dotted black line denotes null model; **(C)** Brier score scaled to the null model (0 variables) or index prediction accuracy (IPA).

**Figure 3:**
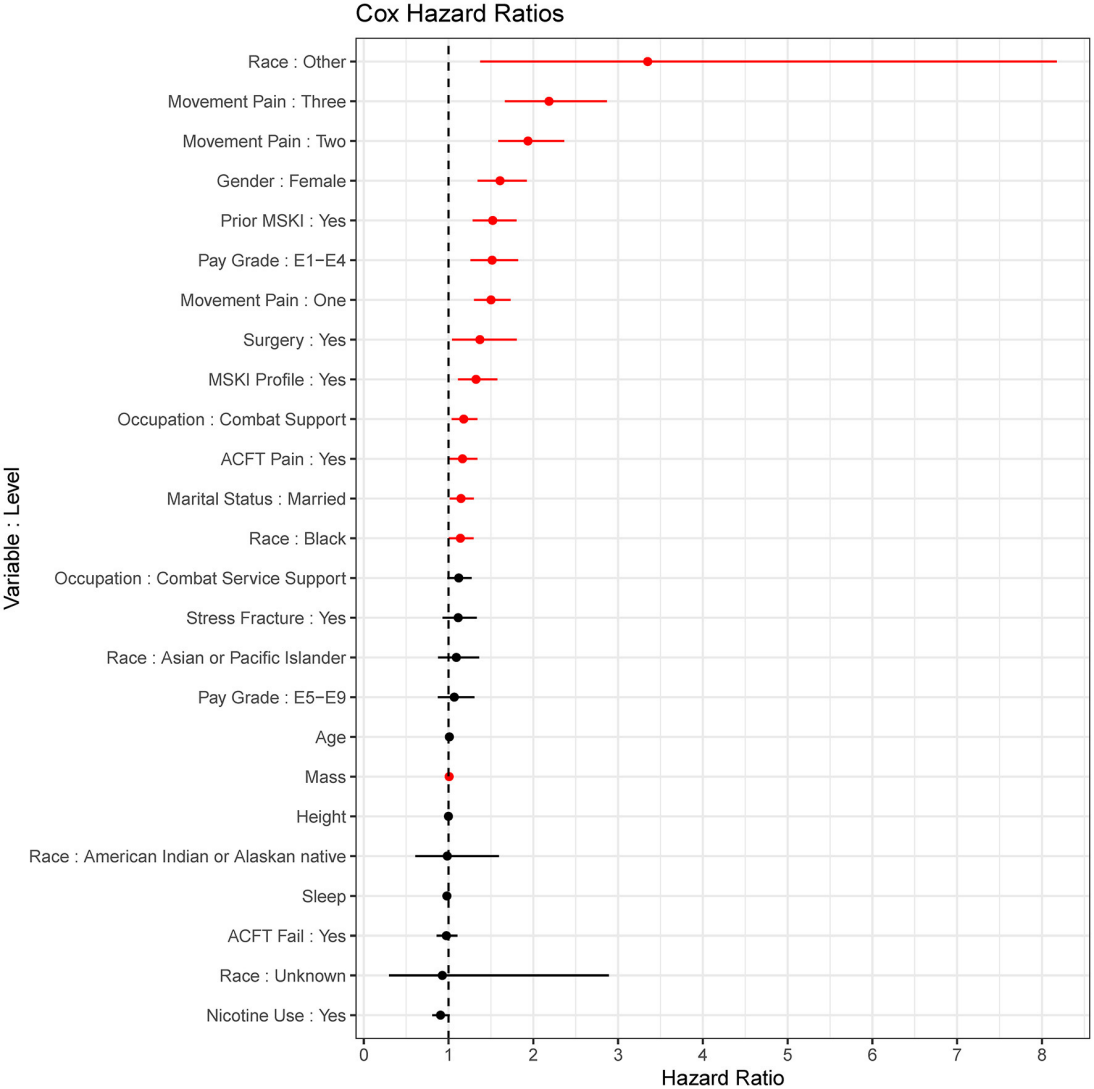
Cox proportional hazards regression (COX) model (final model) coefficients. *Red* = significant (*p* < 0.05), *Black* = non-significant; dots are hazard ratios with bars signifying a 95% confidence interval.

The MSKI incidence rates were 780.9 per 1,000 person-years, 785.1 per 1,000 person-years, and 768.3 per 1,000 person-years for the entire data set (100%), the training data set (75%), and the test data set (25%), respectively, during the 1 year surveillance period. The t-AUC performance for COX, COX-S, and RF were similar at each time horizon, while the CTREE t-AUC was ~0.05 less at each time horizon. There was a 0.06 t-AUC decline from 30 to 365 days averaged across all models (Figure 2A). The absolute Brier score demonstrated similar performance across each model at each time horizon, with no models greater than the null model (0 variables; Figure 2B). The scaled Brier score (i.e., IPA, Figure 2C) was similar across all models up to 90 days (90 days = 10–12%), while the CTREE declined over time, and the COX IPA performance was greater at all time horizons past 90 days compared to the other models. Overall, the COX model had the best performance compared to the COX-S, RF, and CTREE when evaluating the t-AUC and IPA. Based on the criterion at 180 days, the COX model was chosen as the final model for evaluation (t-AUC = 0.70 and IPA = 12.4%) and further analysis. The COX model demonstrated the top five MSKI risk factors were {reported as hazard ratio and 95% confidence interval [HR (95% CI)]}: “other” race [3.3 (1.4–8.2)], three movement pain items [2.2 (1.7–2.9)], two movement pain items [1.9 (1.6–2.4)], female gender [1.6 (1.3–1.9)], and prior MSKI [1.5 (1.3–1.8)] (Figure 3).

**Figure 4:**
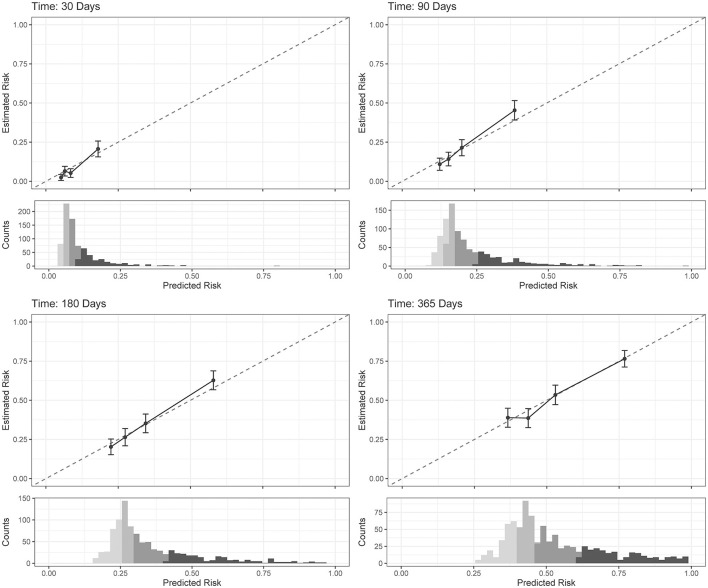
Cox proportional hazards regression (COX) model (final model) calibration curves for each time horizon. Dots represent mean calibration within each risk group bin, with standard error of the counts of subjects across the bin. Histograms represent the distribution of Service member counts in each risk bin across the predicted risk. Histograms shading represent risk bins: light gray = risk bin 1 … black = risk bin 4.

**Figure 5:**
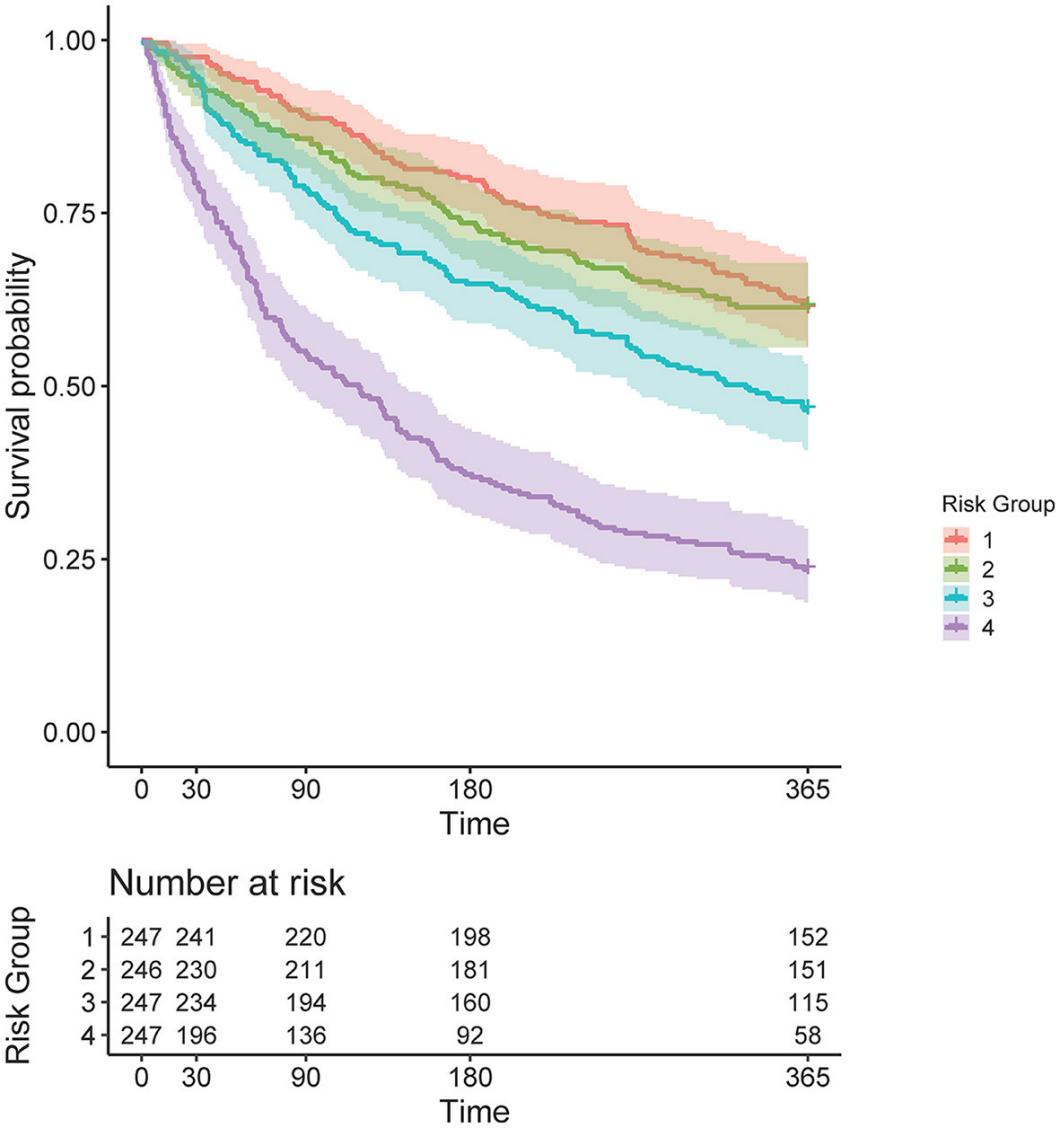
Kaplan–Meier curve, stratified by four risk bins and number of subjects at risk for each time horizon.

The COX model calibration curve binned into quartiles demonstrated four groups of predicted and estimated risk across 30, 90, 180, and 365 days (Figure 4). The estimated risk fell within the mean predicted risk point estimate and standard error, indicating good calibration of the COX final model (Figure 4). The median survival time (95% confidence interval) in risk bin 4 was 119 (85–142) days, in risk bin 3 was 331 (268-NA) days, and for risk bins 1 and 2 could not be calculated within 1 year (Figure 5).

Risk bin 4 had the highest IR and IRR at each time horizon. The overall IR was lowest at the time horizon of 365 days when compared to 30, 90, and 180 days, respective to each risk bin (Table 2). In risk bin 4, compared to risk bin 1, there were a greater proportion of female members, enlisted Service members, Service members with prior MSKIs, Service members with pain during the Army Combat Fitness Test (ACFT), those in combat service support occupations, married Service members, non-white Service members, Service members with an MSKI-related limited duty profile, those with prior stress fracture, those with prior surgery, and those with >1 pain across movement screens (Table 3). In addition, risk bin 4 Service members were shorter in height (bin 4: 173.2 ± 9.4 cm, bin 1: 176.8 ± 7.1 cm; *p* = < 0.001), reported less sleep (bin 4: 6.0 ± 1.1 h, bin 1: 6.6 ± 0.9 h; *p* < 0.001), and were older (bin 4: 25.6 ± 6.4 y, bin 1: 22.9 ± 4.5 y; *p* < 0.001) than risk bin 1 (Table 3). Risk bin 3 had similar findings as risk bin 4 when compared to risk bin 1, while risk bin 2 was more comparable to risk bin 1.

**Table 2:** Risk bins across time horizons incidence rates.

**Time (days)**	**Quantile (risk bin)**	**IR ±SE**	**IRR (95%CI)**	**IRR *p*-value**	**Log rank**	**Log rank *p*-value**
30	1	299.51 ± 122.27	Reference			
	2	818.03 ± 204.51	2.73 (1.07, 6.98)	0.036	4.79	0.029
	3	654.93 ± 181.65	2.19 (0.83, 5.75)	0.113	2.65	0.104
	4	2,817.26 ± 394.5	9.41 (4.04, 21.92)	< 0.001	40.24	< 0.001
90	1	465.73 ± 89.63	Reference			
	2	628.94 ± 106.31	1.35 (0.82, 2.23)	0.241	1.39	0.238
	3	973.8 ± 133.76	2.09 (1.32, 3.32)	0.002	10.36	0.001
	4	2,474.68 ± 234.89	5.31 (3.49, 8.09)	< 0.001	76.10	< 0.001
180	1	449.21 ± 64.17	Reference			
	2	626.32 ± 77.69	1.39 (0.96, 2.02)	0.079	2.79	0.095
	3	891.95 ± 95.63	1.99 (1.4, 2.82)	< 0.001	14.57	< 0.001
	4	2,130.82 ± 171.15	4.74 (3.44, 6.54)	< 0.001	101.22	< 0.001
365	1	480.94 ± 49.34	Reference			
	2	510.57 ± 52.38	1.06 (0.8, 1.41)	0.680	0.12	0.733
	3	789.63 ± 68.73	1.64 (1.26, 2.14)	< 0.001	13.13	< 0.001
	4	1,721.2 ± 125.2	3.58 (2.8, 4.58)	< 0.001	99.50	< 0.001

Risk bins stratified based on the Cox proportional hazards regression model. Incidence rates (IR) ± standard error (SE); Incidence rate ratios (IRR) (95% confidence interval); Reference group (risk bin 1) compared to 2, 3, and 4 risk bins within each time horizon.

**Table 3:** Risk bin descriptive statistics.

**Variable**	**Levels**	**Risk bins**				***P*** **-values**			
		**One (*****n*** = **247)**	**Two (*****n*** = **246)**	**Three (*****n*** = **247)**	**Four (*****n*** = **247)**	**Omnibus**	**One vs. two**	**One vs. three**	**One vs. four**
Paygrade	Officer	26.3%	6.9%	8.9%	10.1%	0.001	0.001	0.001	0.002
	E5-E9	19.4%	16.7%	17.0%	21.1%				
	E1-E4	54.3%	76.4%	74.1%	68.8%				
Occupation	Combat arms	67.6%	32.9%	27.9%	19.8%	0.001	0.001	0.001	0.001
	Combat Service support	18.2%	29.3%	41.7%	48.2%				
	Combat support	14.2%	37.8%	30.4%	32.0%				
Gender	Male	99.6%	96.7%	78.5%	70.9%	0.001	0.668	< 0.001	< 0.001
	Female	0.4%	3.3%	21.5%	29.1%				
Marital status	Single	79.4%	72.4%	59.9%	55.5%	0.001	1.00	< 0.001	< 0.001
	Married	20.6%	27.6%	40.1%	44.5%				
Race	White	89.1%	80.5%	69.6%	53.4%	0.001	0.256	0.001	0.001
	Asian or Pacific Islander	3.2%	8.1%	3.6%	6.5%				
	Black	6.5%	11.4%	24.7%	36.4%				
	American Indian or Alaskan native	0.8%	–	1.6%	0.8%				
	Unknown	0.4%	–	0.4%	–				
	Other	–	–	–	2.8%				
Prior MSKI	No	100.0%	98.4%	89.9%	50.2%	0.001	1.00	< 0.001	< 0.001
	Yes	–	1.6%	10.1%	49.8%				
Nicotine use	No	57.9%	67.5%	76.9%	71.3%	0.005	0.901	0.001	0.120
	Yes	42.1%	32.5%	23.1%	28.7%				
ACFT fail	No	86.6%	85.0%	81.4%	76.1%	0.469	1.00	1.00	0.167
	Yes	13.4%	15.0%	18.6%	23.9%				
ACFT pain	No	99.2%	96.3%	88.3%	57.9%	0.001	0.944	< 0.001	< 0.001
	Yes	0.8%	3.7%	11.7%	42.1%				
Surgery	No	100.0%	99.2%	98.8%	89.1%	0.001	1.00	1.00	< 0.001
	Yes	–	0.8%	1.2%	10.9%				
MSKI profile	No	100.0%	99.2%	91.9%	54.3%	0.001	1.00	< 0.001	< 0.001
	Yes	–	0.8%	8.1%	45.7%				
Stress fracture	No	98.0%	93.9%	95.5%	82.2%	0.001	0.706	1.00	< 0.001
	Yes	2.0%	6.1%	4.5%	17.8%				
Movement pain	0	99.6%	98.4%	83.4%	35.6%	0.001	1.00	0.001	0.001
	1	0.4%	1.6%	14.2%	36.8%				
	2	–	–	2.0%	15.8%				
	3	–	–	0.4%	11.7%				
Age (years)		22.9 ± 4.5	23.1 ± 5.1	24.3 ± 5.1	25.6 ± 6.4	< 0.001	1.00	0.063	< 0.001
Mass (kg)		76.1 ± 10.3	78.7 ± 9.5	79.0 ± 12.4	78.0 ± 13.1	0.668	0.167	0.182	1.00
Height (cm)		176.8 ± 7.1	176.0 ± 7.9	174.5 ± 9.3	173.2 ± 9.4	0.001	1.00	0.112	< 0.001
Sleep (hours)		6.6 ± 0.9	6.6 ± 1.0	6.2 ± 1.0	6.0 ± 1.1	< 0.001	1.00	< 0.001	< 0.001

Descriptive statistics for each risk bin from the Cox proportional hazards regression model; categorical predictors are proportions (%) across the grouping feature, Fisher's exact test across risk bins; numeric features are means ± standard deviation, one-way ANOVA across risk bins; dashed line (–) denotes, “no value”.

## Discussion

Our overall objective was to evaluate if data elements extracted from self-reported questionnaires and existing data sources in military Service members could be used to create a predictive model for assessing MSKI risk. We (1) created a valid and modular MSKI risk predictive model, (2) determined what key features are important for MSKI risk modeling, and (3) provided a framework for MSKI risk stratification through self-reported and existing data sources.

Overall, the models we evaluated were discriminative (t-AUC COX and RF, 30- to 180-day time horizons = 0.73–0.70), and all models performed better than the null model (0 variables) across all time horizons (Figure 2). The COX model demonstrated the best overall performance across all time horizons compared to the other models. However, our models performed worse than previously described military MSKI risk models (Rhon et al., 2022b; Shaw et al., 2023). In an Army Ranger cohort, the logistic regression and cross-validation techniques produced a discriminative (cross-validation AUC = 0.90) and accurate (Brier score = 0.12) MSKI risk model with demographic, biomechanical, and Army physical fitness scores (Rhon et al., 2022b). Similarly, an RF model for medial tibial stress syndrome in military cadets, with an externally validated dataset of Australian Navy recruits, demonstrated discriminative (test set AUC = 0.92) capabilities (Shaw et al., 2023). In general, the worse performance of our models may relate to the granularity of variables being assessed. In our study, dichotomized variables (e.g., yes/no response) were used and may not have provided enough information for the algorithms to “learn” or the variables were not as closely related to MSKI when compared to more granular data (e.g., biomechanical data). Furthermore, previous research did not apply survival-type models; thus, it is difficult to make model performance comparisons (Rhon et al., 2022b; Shaw et al., 2023).

Survival analyses are rarely utilized in MSKI risk research unless for inference and association rather than prediction (e.g., epidemiologic research) (Sharma et al., 2017; Hando et al., 2023). For example (Nielsen et al., 2019a), out of 31 original research studies, only two used “time-to-event” or survival analyses (Drew and Finch, 2016) when associating training load to MSKI outcomes. In MSKI risk research, it may be more advantageous to analyze a rate (e.g., survival–hazard ratios) compared to a static risk (e.g., odds ratios), especially when analyzed over an extended exposure time (e.g., 1 year). By calculating a rate, exposure time is included in the modeling, which could be helpful in determining how useful a screening tool/measurement is up to a specified time horizon, as determined by t-AUC or expected survival time among particular groups (Nielsen et al., 2019a). To a clinician, time-to-event may assist in allocating resources, such as staff or additional screening, to mitigate MSKI risk among a group of Service members before the MSKI occurrence at a specified time horizon (Nielsen et al., 2019b).

Our baseline COX model outperformed all models when evaluating the varying performance metrics. The RF model performed similarly to COX, but the COX model was chosen as the final model to aid clinical interpretation due to the slightly better performance at 180 days (t-AUC 180 days: COX = 0.70, RF = 0.69; IPA 180 days: COX = 12.4%, RF = 10.2%). The COX model may have outperformed the RF model due to the large number of dichotomized variables (RF is robust to continuous type variables but may lead to overfitting) or a few variables explaining the majority of model performance. While the variables most important for modeling (i.e., prior MSKI, female gender, and movement pain; Figure 3) are in agreement with decades of previous research (Rhon et al., 2022a; Hando et al., 2023), many of these are non-modifiable and subject to minimal change states over an extended exposure time (e.g., occupation changes infrequently; Table 1).

Models such as RF are typically deemed “black box” algorithms as there are ensembles of decision trees that are utilized for model building, and extrapolating a single decision tree does not reflect the overall ensemble of trees (Ishwaran et al., 2008; Price, 2018). On the other hand, the CTREE is a single decision tree that is considered a “white box” algorithm and may be easier for a clinician to interpret (Loyola-Gonzalez, 2019). However, the CTREE model had the worst overall model performance (t-AUC 180 days = 0.65). We included the CTREE decision tree (Supplementary Figure 1) and the COX model hazard ratios (Figure 3) to demonstrate the interoperability of these “white box” algorithms for immediate MSKI risk decision support tools.

To empirically evaluate the COX model, Service members in the test data were stratified into quartiles based on predicted risk at four time horizons. In the COX model, when compared to risk bin 1, risk bin 4 had a 9.41 × (30 days), 5.31 × (90 days), 4.74 × (180 days), and 3.58 × (365 days) significantly greater MSKI risk (Table 2). Thus, we successfully stratified groups into low- to high-MSKI risk bins. Our approach may be utilized as a clinical decision support tool to identify Service members who require more in-depth screenings to assess their MSKI risk more thoroughly. Our recommended utilization for the MSKI risk prediction model is to stratify Service members into quartiles or use the top percentage of participants (e.g., top 10%) predicted probability risk to create an “at risk” bin for further screening and potential intervention (Figure 1B) (Roach et al., 2023). An example may be to follow up on the “at-risk” participants with biomechanical assessments that provide feedback to mitigate potential dysfunctional movement patterns (Bird et al., 2022) or in-depth clinical assessments to identify (i.e., Figure 1C: additional screening), properly diagnose (i.e., Figure 1C: Human Performance Staff), and treat present MSKIs (i.e., Figure 1C: Intervention). We stratified the Service members into quartiles, but, dependent on the staffing and time allotted for additional screening and/or interventions, other stratification methods may be used (e.g., quintiles) to increase/decrease the number of Service members who screen into each quantile. Regardless of the number of MSKI risk categories, identifying a “high-risk group” with routinely collected variables (i.e., self-reported MSKI risk screen data = ~30min time to assess 150 Service members) or already collected data (i.e., health records), this may be a method to drastically reduce the number of Service members requiring more in-depth MSKI risk screening (Roach et al., 2023).

Congruent with previous research, we found that female gender, prior MSKI, and pain with movement assessments were MSKI risk factors (Rhon et al., 2022a; Roach et al., 2023). Interestingly, the selection of “other race” had the highest hazard ratio [3.3 (1.4–8.2)] (Figure 3), and Service members who identified as “other” were all distributed to the high-risk bin after stratification (Table 3). All race data were extracted from DEERS and included the following categories: White, Asian or Pacific Islander, Black, American Indian or Alaskan, Other, and Unknown. While several studies have identified an association between race and MSKI risk, the findings are contradictory, and there is no clear association as to which race is at the highest MSKI risk (Sammito et al., 2021). Furthermore, MHS data systems do not fully capture race data, as Service members are unable to view or edit their DEERS data (Michael-Anne Browne, 2023). Thus, we are unable to determine whether disparities exist, and further investigation into race and MSKI risk is needed. On the other hand, modifiable risk factors (e.g., pain with three and two movement assessments) were highly associated with MSKI (Figure 3). Thus, pain with movement assessments may provide a guided in-depth screening to understand the underlying mechanism of pain, allowing a human performance staff team member to potentially intervene (Figure 1).

The primary strength of our study was developing a training model and testing the model on hold-out test data, as many original research articles conclude their findings with the training model (Van Eetvelde et al., 2021). Additionally, we provided potential cut-points to assess MSKI risk for clinicians (Table 3) and a framework for MSKI risk modeling (Figure 1) using existing data sources. However, our study is not without limitations. Our primary limitation is that our analyses include MSKI diagnostic codes, as MSKIs are only identified if the Service member sought care for the MSKI. Service members often do not seek medical care, and MSKIs may go unreported (Sauers et al., 2016). Additionally, ~300 observations were removed due to data being incomplete, which is common when utilizing secondary data sources. Furthermore, our framework only designates high-risk Service members (*n* = 247) for in-depth screening, which inherently leaves 740 Service members without more in-depth MSKI risk screening data. Since our model is based on MSKI risk probability, we decided to structure our framework to further screen Service members at the greatest estimated MSKI risk probability to reduce the time burden on clinicians due to the large number of U.S. military Service members.

## Conclusion

Service members' MSKI risk levels can be determined from self-reported MSKI risk screens and existing military data. This high-throughput approach will improve the ability to complete initial MSKI risk assessment en masse and reduce the number of Service members requiring “in-depth” screenings, as healthcare providers and strength and conditioning professionals can focus their efforts on the highest risk individuals. Decreasing additional screenings across all Service members reduces the time burden on the Service member and clinical staff and increases resource allocation to other necessities across the MHS. Thus, the next step is to deploy this type of algorithm directly into an electronic health record or within an operational military unit. This original research is a continuation of the modernization of the MHS with 21st-century capabilities by delivering a framework that may be deployable in the MHS infrastructure.

## Data availability statement

The datasets presented in this article are not readily available because the datasets generated and analyzed during the current study are not publicly available because of data sharing restrictions on data generated within the United States Department of Defense; however, data may be available from the corresponding author on reasonable request, following approval from all required regulatory bodies. Requests to access the datasets should be directed to MB, matthew.b.bird.civ@health.mil.

## Ethics statement

This study received an exempt determination as non-human subject research by the Womack Army Medical Center Human Research Protections Office (Protocol #22-14804; Approved 14 June 2022).

## Author contributions

MB: Conceptualization, Data curation, Formal analysis, Investigation, Methodology, Validation, Visualization, Writing – original draft, Writing – review & editing. MR: Conceptualization, Data curation, Investigation, Methodology, Writing – original draft, Writing – review & editing. RN: Formal analysis, Methodology, Validation, Visualization, Writing – original draft, Writing – review & editing. MH: Conceptualization, Investigation, Writing – original draft, Writing – review & editing. TM: Conceptualization, Data curation, Investigation, Methodology, Project administration, Supervision, Writing – original draft, Writing – review & editing.

## Supplementary material

Supplementary Figure 1 Random forest (RF) variable importance.

Supplementary Figure 2 Conditional inference trees (CTREE).
