# Supplementary material for: A machine learning framework to classify musculoskeletal injury risk groups in military service members
Source: Front Artif Intell. 2024 Jun 19;7:1420210. doi: 10.3389/frai.2024.1420210 (PMC11325721; doi:10.3389/frai.2024.1420210)
Supplement: Supplementary file 1 [file Data_Sheet_1.PDF]

Prediction Survival Script for the Manuscript:

A Machine Learning Framework to Classify Musculoskeletal Injury Risk Groups in Military Service Members

Matthew B. Bird, PhD, CSCS[1, 2*], Megan H. Roach, PhD, ATC[1, 2, 3], Roberts G. Nelson MS[4], Matthew S. Helton, PT, DSc[5], Timothy C. Mauntel, PhD, ATC[1, 2, 3]

[1]Extremity Trauma & Amputation Center of Excellence, Defense Health Agency, Falls Church, VA, USA, [2]Department of Clinical Investigations, Womack Army Medical Center, Fort Liberty, NC, USA, [3]Department of Surgery, Uniformed Services University of the Health Sciences, Bethesda, MD, USA, [4]Artificial Intelligence Task Force, Army Futures Command, Pittsburgh, PA, [5]U.S. Army, Tripler Army Medical Center, Honolulu, HI, USA

Packages

```
library(openxlsx)
library(dplyr)
library(readr)
library(knitr)
library(forcats)
library(ggfortify)
library(gridExtra)
library(survminer)

library(caret)
library(riskRegression)
library(tidymodels)

library(survival)
library(ranger)
library(gbm)
library(splines)
library(party)

library(flextable)
library(stringr)

set.seed(0)
```

Data Preparation

Load the Data

Load the raw data.

```
#Read in data
raw_data<- read.xlsx("data.xlsx", detectDates = TRUE)

#Count NAs in columns
na_count <-sapply(raw_data, function(y) sum(length(which(is.na(y)))))
  data.frame(na_count) %>%
    mutate(total_n = 4222)%>% #fill in total observations
    mutate(missing = na_count/total_n)

#Drop NAs for the variables to be used in analysis (Complete cases)
```

```
raw_data<-raw_data %>%
    drop_na(Occupation) %>%
    drop_na(Pay_Grade) %>%
    drop_na(Race) %>%
    drop_na(Height)
```

Pre-Process the Data

Identify the factor and numeric covariates.

```
factor_cols <- c(
  "Pay_Grade",
  "Occupation",
  "Gender",
  "Marital_Status",
  "Race",
  "Prior_Mski",
  "Nicotine_Use",
  "ACFT_Fail",
  "ACFT_Pain",
  "Surgery",
  "MSKI_Profile",
  "Stress_Fracture",
  "Movement_Pain"
)

numeric_cols <- c(
  "Age",
  "Mass",
  "Height",
  "Sleep",
  "all_encounter_date",
  "all_msk"
)

numeric_covariates <- numeric_cols[! numeric_cols %in% c("all_encounter_date", "all_msk")]

data <- raw_data |>
  select(all_of(c(factor_cols, numeric_cols))) |>
  mutate_at(factor_cols, factor)
```

Rename factor levels for cleaning purposes

```
data <- data %>%
  mutate(
    Race = recode(
      Race,
      "C" = "White",
      "M" = "Asian or Pacific Islander",
      "N" = "Black",
      "R" = "American Indian or Alaskan native",
      "X" = "Other",
      "Z" = "Unknown"
    ),
    Marital_Status = recode_factor(Marital_Status,
                                   "S" = "Single",
                                   "M" = "Married"),
    Marital_Status = fct_relevel(Marital_Status, "Single", "Married"),
    Gender = recode(Gender,
                    "M" = "Male",
                    "F" = "Female"),
    Gender = fct_relevel(Gender, c("Male", "Female")),
```

```
    Pay_Grade = fct_relevel(Pay_Grade, c('Officer' , "E5-E9", 'E1-E4'))
  )
```

Create surveillance cut-point at one-year

```
data <-
  survSplit(Surv(all_encounter_date, all_msk) ~ ., data = data, cut = 365.25,
                episode="epoch") |>
  subset(epoch == 1) |>
  select(-c(epoch,tstart))
```

Holdout Data

Create a training set and holdout test set in a 75%/25% split.

```
train_idx <- createDataPartition(data$all_msk,
                                 times = 1,
                                 p = 0.75,
                                 list = FALSE)

train_data <- data[train_idx, ]
test_data <- data[-train_idx, ]
```

EDA

Plot Kaplan-Meier curves for categorical variables such as sex.

```
km <- survfit(Surv(all_encounter_date, all_msk) ~ Gender, data=train_data)
autoplot(km)
```

Train Models

Baseline Model: Cox Proportional Hazards

The baseline model is a simple Cox Proportional Hazards model.

```
cox <- coxph(
  Surv(all_encounter_date, all_msk) ~ .,
  x=TRUE,
  data=train_data
)
ggforest(cox)
```

Candidate Model: Cox with Splines

Sometimes we can improve on the Cox PH model by adding flexibility to the covariates through natural (restricted cubic) splines. We let each of the numeric features have a basis of natural splines with varying number of internal knots and select the model with the lowest Akaike information criterion (AIC).

```
#Enter covariates in function
fit_cox_with_spline <- function(k_age, k_mass, k_height, k_sleep) {
  model <- coxph(
    Surv(all_encounter_date, all_msk) ~
      Pay_Grade + Occupation + Gender + Marital_Status + Race +
      Prior_Mski + Nicotine_Use + ACFT_Fail + ACFT_Pain +
      Surgery + MSKI_Profile + Stress_Fracture + Movement_Pain +
      ns(Age, k_age) + ns(Mass, k_mass) +
      ns(Height, k_height) + ns(Sleep, k_sleep),
    x = TRUE,
```

```
    data = train_data
  )
}

#Number of potential knots for each covariate
spline_grid <- expand_grid(
  k_age = c(1, 2, 3, 4, 5),
  k_mass = c(1, 2, 3, 4, 5),
  k_height = c(1, 2, 3, 4, 5),
  k_sleep = c(1, 2, 3, 4, 5)
)

spline_results <- spline_grid

spline_results <- spline_results %>%
  mutate(AIC = pmap_dbl(., \(...) fit_cox_with_spline(...) |> AIC()))

spline_results
```

Get knot results for each covariate based on lowest AIC value

```
spline_results |>
  slice_min(AIC)
```

Unfortunately, riskRegression::Score uses the coxph function call, so you need to manually define the spline degrees of freedom.

```
cox_spline <- coxph(
    Surv(all_encounter_date, all_msk) ~
      Pay_Grade + Occupation + Gender + Marital_Status + Race +
      Prior_Mski + Nicotine_Use + ACFT_Fail + ACFT_Pain +
      Surgery + MSKI_Profile + Stress_Fracture + Movement_Pain +
      ns(Age, 1) + ns(Mass, 1) + #Enter in the number of knots from previous result for each covariate
      ns(Height, 2) + ns(Sleep, 2),
    x = TRUE,
    data = train_data
  )
```

We can compare the two models with ANOVA to determine how much the spline helped the model fit compared to linear terms.

```
anova(cox, cox_spline)
```

It can be useful to look at the individual term plots from the model to see the effect of the non-linearity.

```
termplot(
  cox_spline,
  se = TRUE,
  col.se = "darkblue",
  col.term = "black",
  ylim = "free"
)
```

Candidate Model: Random Survival Forest

The next candidate model is a random survival forest. This is a fully non-parametric model that does not rely on assumptions like proportional hazards.

Run a grid search over `mtry` and `min.node` to control the bias and variance of the forest and individual trees and use out-of-bag prediction error to estimate each surrogate model's performance on unseen data.

```r
num_trees <- 100

mtry_grid <- c(2, 4, 6, 8, 10)
node_grid <- c(3, 5, 7, 9)

rsf_models <- crossing(
  mtry=mtry_grid,
  min_node_size=node_grid
)

fit_rsf <- function(mtry, min_node_size) {
  rsf <- ranger(
    Surv(all_encounter_date, all_msk) ~ .,
    mtry=mtry,
    min.node.size=min_node_size,
    num.trees=num_trees,
    data=train_data,
    respect.unordered.factors=TRUE
  )
  return(rsf$prediction.error)
}

rsf_models <- rsf_models |>
  mutate(
    prediction_error = map2(mtry, min_node_size, fit_rsf) |> as.numeric()
  )
```

We can visualize the results of the cross-validation.

```r
ggplot(
  data=rsf_models,
  aes(
    x=mtry,
    y=prediction_error,
    color=factor(min_node_size)
  )
) +
  geom_point() +
  geom_line() +
  theme_bw()
```

Pick the set of hyperparameters that minimized prediction error and fit the final model.

```r
num_trees <- 500

best_rsf_parameters <- rsf_models |>
  slice_min(prediction_error) #Automatically tuned on the results from previous chunk

rsf <- ranger(
  Surv(all_encounter_date, all_msk) ~ .,
  mtry=best_rsf_parameters$mtry,
  min.node.size=best_rsf_parameters$min_node_size,
  num.trees=num_trees,
  data=train_data,
  importance="permutation",
  scale.permutation.importance=TRUE,
  keep.inbag=TRUE,
  respect.unordered.factors=TRUE
)
```

k- fold cross-validation

Run this chunk before the next Explanatory Model (CTREE)

k-fold cross-validation over interaction depth and the minimum number of observations per node.

```
# custom code for k-fold with a param grid
kfold <- function(param_grid, folds, fit_surrogate, score_fold) {
  result <- c()

  fit_score_fold <- function(splits, id) {
    train <- analysis(splits)
    validation <- assessment(splits)

    fold_score <- param_grid
    surrogate_models <- pmap(fold_score, fit_surrogate, train=train)
    fold_score$score <- map_dbl(surrogate_models, score_fold, validation=validation)
    fold_score$id <- id

    fold_score
  }

  result <- pmap(folds, fit_score_fold)

  bind_rows(result)
}

#Evaluate model on 180 days
score_fold <- function(model, validation, eval_time=180) {
  score <- Score(
    list(
      "model"=model
    ),
    formula=Surv(all_encounter_date, all_msk) ~ 1,
    data=validation,
    times=c(eval_time)
  )$AUC$score |>
    tibble() |>
    select(AUC) |>
    pull()
}

folds <- vfold_cv(train_data, v = 10)
```

Explanatory Model - Conditional Tree

In cases where model explainability is important, simple decision trees can be powerful. They are easy to understand and explain to people with little or no knowledge of statistical models.

```
ctree_grid <- expand_grid(
  mincriterion=c(0.99, 0.95, 0.9, 0.85, 0.8)
)

fit_ctree_surrogate <- function(train, mincriterion) {
  control <- party::ctree_control(mincriterion=mincriterion)
  ctree <- Ctree(
    Surv(all_encounter_date, all_msk) ~.,
    data=train,
    control=control
  )
}

#kfold function created in previous chunk
ctree_cv_result <- kfold(ctree_grid, folds, fit_ctree_surrogate, score_fold)
```

```
mean_ctree_cv_result <- ctree_cv_result |>
  group_by(across(c(-id, -score))) |>
  summarize(
    mean = mean(score),
    se = sd(score)/sqrt(n())
  ) |>
  ungroup()

ggplot(
  mean_ctree_cv_result,
  aes(
    x=mincriterion,
    y=mean
  )
) +
geom_point() +
geom_errorbar(aes(ymin=mean-se, ymax=mean+se)) +
ylab("Mean KFold Score") +
theme_bw()

best_ctree_parameters <- mean_ctree_cv_result |>
  slice_max(mean)

control <- party::ctree_control(mincriterion=best_ctree_parameters$mincriterion)
ctree <- Ctree(
  Surv(all_encounter_date, all_msk) ~.,
  data=train_data,
  control=control
)
```

Evaluate

Score

Score the models across different time horizons from 30 days to 1 year.

```
plot_times <- c(30, 90, 180, 365)
times <- 30:365

score<-Score(
  list(
    "Cox"=cox,
    "Cox Spline"=cox_spline,
    "Random Forest"=rsf,
    "ctree"=ctree
  ),
  formula=Surv(all_encounter_date,all_msk)~1,
  data=test_data,
  times=times,
  plots="calibration",
  summary="ipa"
)
```

Discrimination

Use time-dependent AUC-ROC to measure model discrimination at different time horizons.

```
AUC_ROC_Graph <- tibble(score$AUC$score) %>%
  mutate(Model = as.factor(model)) %>%
  ggplot(.,
```

```
        aes(x = times,
            y = AUC,
            color = Model)) +
  geom_line() +
  xlab("Time [days]") +
  ylab("Time-Dependent AUC-ROC") +
  theme_bw() +
  scale_color_manual(
    values = c(
      "Cox" = "darkgreen",
      "Cox Spline" = "purple",
      "ctree" = "red",
      "Random Forest" = "blue"),
    labels = c('COX', "COX-S", "RF", "CTREE")
  )

AUC_ROC_Graph

#Clean the AUC_ROC graph
AUC_ROC_Graph_y <-
  AUC_ROC_Graph +
  scale_y_continuous(breaks = seq(.6, .75, .05), limits = c(.6, .75))  + scale_x_continuous(breaks=c(30,90,180
,365), limits = c(30, 365))#Manipulate y-axis limits

AUC_ROC_Graph_y

#Prints the AUC score at each time horizon
AUC_Scores_df<-score$AUC$score |>
          filter(times == 30 | times == 90 | times == 180 | times == 365)

AUC_Scores_df
```

Brier Score

Use the Brier score to evaluate overall model performance.

```
Brier_Score_Graph <- tibble(score$Brier$score) %>%
  mutate(Model = as.factor(model)) %>%
  ggplot(.,
        aes(
          x = times,
          y = Brier,
          color = Model,
          linetype = Model
        )) +
  geom_line() +
  xlab("Time [days]") +
  ylab("Brier Score") +
  theme_bw() +
  scale_linetype_manual(values = c(
    "Null model" = 2,
    "Cox" = 1,
    "Cox Spline" = 1,
    "ctree" = 1,
    "Random Forest" = 1
  )) +
  scale_color_manual(
    values = c(
      "Null model" = "black",
      "Cox" = "darkgreen",
      "Cox Spline" = "purple",
      "ctree" = "red",
      "Random Forest" = "blue"),
```

```
    labels = c("Null Model",'COX', "COX-S", "RF", "CTREE")
  )


Brier_Score_Graph

#Clean the Brier graph
Brier_Score_Graph_y <-
  Brier_Score_Graph + scale_y_continuous(breaks = seq(.05, .25, .05), limits = c(.05, .25))  + scale_x_continu
ous(breaks=c(30,90,180,365), limits = c(30, 365))  #Manipulate y-axis limits

Brier_Score_Graph_y

#Prints the Brier score at each time horizon
Brier_Score_df<-score$Brier$score |>
              filter(times == 30 | times == 90 | times == 180 | times == 365)

Brier_Score_df
```

Index of Prediction Accuracy (Scaled Brier Score)

The scaled Brier score is easier to interpret as a percentage increase in performance over a null model.

```
IPA_Graph <- tibble(summary(score)$score) %>%
  mutate(IPA = as.numeric(IPA)) %>%
  filter(Model != "Null model") %>%
  ggplot(.,
         aes(x = times,
             y = IPA,
             color = Model)) +
  geom_line() +
  xlab("Time [days]") +
  ylab("Scaled Brier Score") +
  theme_bw() +
  scale_color_manual(
    values = c(
      "Cox" = "darkgreen",
      "Cox Spline" = "purple",
      "ctree" = "red",
      "Random Forest" = "blue"),
    labels = c('COX', "COX-S", "CTREE", "RF")
  )


IPA_Graph

#Clean the IPA graph
IPA_Graph_y <-
  IPA_Graph + scale_y_continuous(breaks = seq(4, 14, 2), limits = c(4, 14)) + scale_x_continuous(breaks=c(30,9
0,180,365), limits = c(30, 365))

IPA_Graph_y

#Prints the IPA score at each time horizon
IPA_Scores_df <-summary(score)$score |>
                filter(times == 30 | times == 90 | times == 180 | times == 365) |>
                select(c(times, Model, IPA))

IPA_Scores_df
```

Combine Scaled Brier and AUC-ROC Graphs

```
Performance_Graphs_Combined <-
  ggarrange(
    AUC_ROC_Graph_y,
    Brier_Score_Graph_y,
    IPA_Graph_y,
    labels = c("A", "B", "C"),
    ncol = 1,
    common.legend = TRUE,
    legend = "bottom"
  )

Performance_Graphs_Combined

#Save the graphs in one graph
ggsave(
  file = "Performance_Graphs_Combined.jpeg",
  dpi = 400,
  height = 10,
  width = 8
)



#Prints the AUC, Brier, and IPA score at each time horizon
Total_Scores_df <-summary(score)$score |>
                filter(times == 30 | times == 90 | times == 180 | times == 365)

Total_Scores_df
```

Model Selection

Select model that produces the best (lowest) Brier score at 180 days.

```
model_list <-
  list(
    "Cox"=cox,
    "Cox Spline"=cox_spline,
    "Random Forest"=rsf,
    "ctree"=ctree
  )

selection_time <- 180


best_model_name <-
  tibble(score$Brier$score) |>
  filter(times == selection_time) |>
  slice(which.min(Brier)) |>
  pull(model) |>
  as.character()


final_model <- model_list[[best_model_name]]
```

Risk Groups

Cumulative Incidence

For whichever model was determined to be best in the evaluation, we can look at how well it classifies observations into risk groups. This is a more discrete view of the calibration plots.

Here is an implementation from the riskRegression package.

```
# set how many risk groups to make
q <- 4
```

First we compare calibration with bar charts.

```
risks <- predictRisk(final_model,
                     times = plot_times,
                     newdata = test_data) |>
  as_tibble()

names(risks) <- as.character(plot_times)
risks <- risks |>
  mutate(surv = with(test_data, Surv(all_encounter_date, all_msk)))

risks <- risks |>
  rowid_to_column("ID") |>
  pivot_longer(cols = -c(surv, ID),
               names_to = "time",
               values_to = "predicted_risk") |>
  mutate(time = as.numeric(time))

calculate_group_risks <- function(risks, t, q) {
  risks |>
    filter(time == t) |>
    mutate(risk_group = cut(
      predicted_risk,
      breaks = quantile(predicted_risk,
                        seq(0, 1, length.out = q + 1)),
      right = FALSE,
      include.lowest = TRUE
    )) |>
    group_by(risk_group) |>
    summarize(
      mean_predicted_risk = mean(predicted_risk),
      mean_estimated_risk = 1 - (survfit(surv ~ 1) |> summary(times = t))$surv
    )
}

calculate_risk_groups <- function(risks, t, q) {
  risks |>
    filter(time == t) |>
    mutate(risk_group = cut(
      predicted_risk,
      breaks = quantile(predicted_risk,
                        seq(0, 1, length.out = q + 1)),
      right = FALSE,
      include.lowest = TRUE
    ))
}

calculate_group_risks <- function(risks, t) {
  risks |>
    filter(time == t) |>
    group_by(risk_group) |>
    summarize(
      mean_predicted_risk = mean(predicted_risk),
      estimated_risk = 1 - (survfit(surv ~ 1) |> summary(times = t))$surv,
      estimated_risk_se = (survfit(surv ~ 1) |> summary(times = t))$std.err,
      time = t
    )
}

risks <-
  map(unique(risks$time), \(t) calculate_risk_groups(risks, t, q)) |>
```

```r
  bind_rows()

group_risks <-
  map(unique(risks$time), \(t) calculate_group_risks(risks, t)) |>
  bind_rows()

reformat_cut_labels <- function(labels) {
  labels |>
    strsplit(",") |>
    map(parse_number) |>
    map(\(x) round(x*100, 2)) |>
    unlist() |>
    paste(collapse="-")
}

levels(group_risks$risk_group) <-
  map(levels(group_risks$risk_group), reformat_cut_labels) |>
  unlist()

group_risks |>
  rename(Predicted = mean_predicted_risk,
         Estimated = estimated_risk,
         Estimated_SE = estimated_risk_se,
         `Risk Group` = risk_group) |>
  pivot_longer(
    cols=c(Predicted, Estimated),
    names_to="Type",
    values_to="Risk"
  ) |>
  mutate(Estimated_SE = ifelse(Type == "Estimated",
                               Estimated_SE, NA),
         Type = factor(Type, levels = c("Predicted", "Estimated"))) |>
  group_by(time) |>
ggplot(aes(x=`Risk Group`,
           y=Risk,
           fill=Type)) +
  geom_bar(stat="identity", position="dodge") +
  geom_errorbar(aes(ymin = Risk - 1.96*Estimated_SE,
                    ymax = Risk + 1.96*Estimated_SE),
                position = position_dodge(.9),
                width = 0.4) +
  facet_wrap(vars(time), nrow=2, scales="free_x") +
  theme_bw() +
  theme(legend.title=element_blank()) +
  scale_fill_grey()
```

The predictions come from the model and the estimated risk is one minus the Kaplan-Meier estimate.

Here is a visualization of this calibration at different time steps.

```r
group_risks |>
  mutate(
    Time = as.factor(time)
  ) |>
ggplot(aes(x=mean_predicted_risk,
           y=estimated_risk,
           color=Time)) +
  geom_line(linetype="solid") +
  geom_point() +
  geom_errorbar(aes(ymin = estimated_risk - 1.96*estimated_risk_se,
                    ymax = estimated_risk + 1.96*estimated_risk_se),
                width = 0) +
  geom_abline(alpha=0.5, linetype="dashed") +
  xlim(0, 1) +
  ylim(0, 1) +
```

```
  xlab("Mean Predicted Risk") +
  ylab("Estimated Risk") +
  theme_bw()
```

Neither of the two previous plots display information about the distribution of data in each risk bin. The following plot adds this information through a marginal histogram.

```
p<-list()
for (t in plot_times) {
  group_plot <-
    ggplot(group_risks |> filter(time==t)) +
      geom_point(aes(x=mean_predicted_risk,
                     y=estimated_risk)) +
      geom_line(aes(x=mean_predicted_risk,
                     y=estimated_risk)) +
      geom_errorbar(aes(x = mean_predicted_risk,
                        ymin = estimated_risk - 1.96*estimated_risk_se,
                        ymax = estimated_risk + 1.96*estimated_risk_se),
                    width = 0.02) +
      xlim(0, 1) +
      ylim(0, 1) +
      geom_abline(linetype="dashed", alpha=0.5) +
      xlab("Predicted Risk") +
      ylab("Estimated Risk") +
      ggtitle(paste0("Time: ", t, " Days")) +
      theme_bw() +
      theme(axis.title.x = element_blank(), axis.text.x = element_blank())


  individual_plot <-
    ggplot(data=risks |> filter(time==t),
           aes(x=predicted_risk,
               fill=risk_group)) +
    geom_histogram(position="identity", bins=50) +
    xlab("Predicted Risk") +
    ylab("Counts") +
    xlim(0, 1) +
    theme_bw() +
    scale_fill_grey(start=0.8, end=0.2) +
    guides(fill="none") +
    theme(axis.title.y = element_text(margin = margin(t = 0, r = 9, b = 0, l = 0)))


  p[[t]]<-grid.arrange(group_plot, individual_plot, nrow=2, heights=c(1, 0.5))
}

#Arrange plots in one figure
figure_1<-grid.arrange(p[[30]],p[[90]], p[[180]], p[[365]] , nrow =2, ncol = 2)

figure_1

#Save the calibration curve with distribution across time horizons
ggsave(file = "calibration_time_horizons.jpeg", figure_1, dpi = 300, height = 10, width = 12)
```

Use this to add risk group information back to test data.

```
test_data_with_risks <-
  risks |>
    pivot_wider(names_from = time,
                values_from = c(risk_group, predicted_risk, surv)) |>
    mutate(
      surv = !! sym((paste0("surv_", plot_times[[1]])))
    ) |>
    droplevels() |>
    select(-c(map_chr(plot_times, \(x) paste0("surv_", x)))) |>
  bind_cols(test_data)
```

```r
# test that the join happened correctly
with(test_data_with_risks,
     (as.character(surv) == as.character(Surv(all_encounter_date, all_msk))) |>
       all()) |>
  ifelse("The join matches", "There was an error") |>
  print()

print(test_data_with_risks)
```

Incidence Rate

We compare the stratified risk groups by their incidence rates.

Incidence Rate

```r
risk_groups <- map_chr(plot_times, \(x) paste0("risk_group_", x))


calculate_incidence_rate <- function(data, risk_group_str) {
  time <- parse_number(risk_group_str)

  data |>
    group_by(!! sym(risk_group_str)) |>
    summarize(event_count = sum(ifelse(time > all_encounter_date,
                                       all_msk,
                                       0)),
              person_time = sum(pmin(all_encounter_date, time)),
              incidence_rate = (event_count / person_time) * 365.25 * 1000) |>
    ungroup() |>
    rowid_to_column("quantile") |>
    mutate(time = time,
           risk_group = !!sym(risk_group_str),
           quantile = as.factor(quantile),
           incidence_rate_se = sqrt((event_count / person_time^2) * (365.25 * 1000)^2)) |>
    select(-any_of(risk_group_str))
}

incidence_rates <-
  map_df(risk_groups, \(x) calculate_incidence_rate(test_data_with_risks, x))

print(incidence_rates)
```

Incidence Rate Ratio

Now we compare all risk groups to the lowest risk group as a baseline.

```r
incidence_rate_ratio <-
  incidence_rates |>
  group_by(time) |>
  mutate(baseline_rate = incidence_rate[quantile == 1],
         rate_ratio = incidence_rate / baseline_rate,
         log_rate_ratio = log(rate_ratio),
         log_rate_ratio_sem = sqrt(1/event_count + 1/event_count[quantile==1]),
         t = log_rate_ratio / log_rate_ratio_sem,
         pvalue = pnorm(t, lower.tail = FALSE) * 2,
         rate_ratio_95ci_upper = exp(log_rate_ratio + 1.96 * log_rate_ratio_sem),
         rate_ratio_95ci_lower = exp(log_rate_ratio - 1.96 * log_rate_ratio_sem),
         time = time) |>
  ungroup() |>
  relocate(time)

print(incidence_rate_ratio)

#Create a flex table for usable format
incidence_rate_table<-incidence_rate_ratio %>%
```

```
                        select(1:2,5,7)
incidence_rate_table_flex<-incidence_rate_table %>%
  flextable() %>%
  set_caption(
    caption = as_paragraph(
              as_chunk("Incidence Rate", props = fp_text_default( bold = TRUE)))) %>%
  add_footer_lines("Table x: Incidence rate (IR) comparing across quantiles") %>%
  colformat_double(j = c(3,4), digits = 2) #%>%

incidence_rate_table_flex

#Save flex table
incidence_rate_table_flex %>% save_as_docx( path = "incidence_rate.docx")
```

Rate Ratio Cleaning (Extract information from previous chunk)

```
incidence_rate_ratio_summary <-
  incidence_rate_ratio |>
  filter(quantile != 1) |>
  mutate(rr_lower_95 = rate_ratio_95ci_lower,
         rr_upper_95 = rate_ratio_95ci_upper,
         p_value_irr = pvalue) |>
  select(time, quantile, rate_ratio, rr_lower_95, rr_upper_95, p_value_irr)

print(incidence_rate_ratio_summary)
```

Incident Rate Ratio Graph

```
IRR<-incidence_rate_ratio |>
  filter(quantile != 1) |>
  ggplot(aes(x = quantile,
             y = rate_ratio)) +
  facet_wrap(vars(time))+
  xlab("Risk Group Quantile") +
  ylab("Incidence Rate Ratio") +
  geom_point() +
  geom_errorbar(aes(ymin = rate_ratio_95ci_lower,
                    ymax = rate_ratio_95ci_upper)) +
  geom_hline(yintercept = 1, linetype = "dashed") +
  theme_bw()+
  scale_y_continuous(breaks  = seq(0,18, 3))



IRR

#Clean the figure
IRR + facet_wrap(~time, labeller = labeller(time = c("30" = "30 days", "90" = "90 days", "180" = "180 days",
"365" = "365 days")))

#Save the figure
ggsave(file = "IRR.jpeg", dpi = 300)
```

Log-Rank Test

Calculate log-rank between all risk groups and the lowest risk group. Because this generates multiple comparisons (equal to the number of quantiles minus one), a correction is applied to the p-values later.

```
two_way_log_rank <- function(surv, risk_group, reference, treatment) {
  keep_idx <- (risk_group == reference | risk_group == treatment)

  difference <- survdiff(surv[keep_idx] ~ risk_group[keep_idx])
  result <- list(chisq = difference$chisq,
```

```
                 pvalue = difference$pvalue)
}

log_rank <- function(surv, risk_group) {
  # order into quantiles 1 to m
  risk_group <- as.numeric(risk_group)

  group_levels <- seq(2, max(risk_group), 1)
  result <-
    map_df(group_levels,
           \(x) two_way_log_rank(surv, risk_group, reference = 1, treatment = x)) |>
    mutate(quantile = group_levels) |>
    relocate(quantile)
}

log_rank_with_censoring <- function(data, risk_group) {
  results <-
    data |>
    mutate(
      time = parse_number(risk_group),
      eventtime = ifelse(all_encounter_date > time,
                         time, all_encounter_date),
      status = ifelse(all_encounter_date > time,
                      0, all_msk),
      surv_censored = Surv(eventtime, status)
    )

  log_rank(results$surv_censored, results |> pull(risk_group)) |>
    mutate(time = parse_number(risk_group),
           quantile = as.factor(quantile))
}

log_rank_results <-
  map_df(risk_groups,
     \(x) log_rank(test_data_with_risks$surv, test_data_with_risks |> pull(x)) |>
       mutate(time = parse_number(x),
              quantile = as.factor(quantile))
     ) |>
  relocate(time, .before = quantile)
print(log_rank_results)

log_rank_results_with_censoring <-
  map_df(risk_groups, \(x) log_rank_with_censoring(test_data_with_risks, x)) |>
  relocate(time, .before = quantile)
print(log_rank_results_with_censoring)
```

Combine Rate Ratios and Log-Rank

```
risk_group_summary <-
  left_join(incidence_rate_ratio_summary, log_rank_results_with_censoring |>
              mutate(chisq_logrank = chisq,
                     p_value_logrank = pvalue) |>
              select(time, quantile, chisq_logrank, p_value_logrank),
            by = c("time", "quantile"))

print(risk_group_summary)
```

Correct p-values for multiple comparisons.

```
decorate_significance <- function(p) {
  cut(p, breaks = c(0, 0.001, 0.01, 0.05, 0.1, 1)) |>
    as.numeric() |>
    switch("***", "**", "*", ".", " ")
}
```

```
risk_group_summary |>
  mutate(
    p_value_irr = p.adjust(p_value_irr, method = "holm"),
    p_value_logrank = p.adjust(p_value_logrank, method = "holm"),
    significance = map_chr(p_value_logrank, decorate_significance)
  ) |>
  select(time,
         quantile,
         rate_ratio,
         p_value_irr,
         p_value_logrank,
         significance)



risk_group_summary

#Push into flex table for usable format
risk_group_table <- risk_group_summary |>
  flextable() |>
  set_caption(caption = as_paragraph(as_chunk("Incidence Rate Ratio", props = fp_text_default(bold = TRUE))))
|>
  add_footer_lines(
    "Table x: Incidence rate ratios (IRR) comparing across quantiles within risk bins to refrence[risk bin 1]
"
  ) %>%
  colformat_double(j = c(3:5, 7), digits = 2) |>
  colformat_double(j = c(6, 8), digits = 3)

risk_group_table

#Save flex table
risk_group_table |> save_as_docx(path = "risk_group_table.docx")
```

Combine Incidence Rates and Risk Ratios for a finalized table

```
IRR_IR_table <-
  full_join(incidence_rate_table,
            risk_group_summary,
            by = c("time" , "quantile")) |>
  mutate_at(c(3:7, 9), round, 2) |>
  mutate_at(c(8, 10), round, 3) |>
  mutate(incidence_rate = paste0(incidence_rate, "±", incidence_rate_se)) |>
  mutate(rate_ratio = paste0(rate_ratio, "[", rr_lower_95, ",", rr_upper_95, "]")) |>
  select(-c(incidence_rate_se, rr_lower_95, rr_upper_95)) |>
  mutate(rate_ratio = case_when(rate_ratio == "NA[NA,NA]" ~ "Reference",
                                TRUE ~ rate_ratio)) |>
  rename(
    "IR±SE" = "incidence_rate",
    "IRR[95%CI]" = "rate_ratio",
    "IRR p value" = "p_value_irr",
    "Log rank" = "chisq_logrank",
    "Log rank p value" = "p_value_logrank"
  )

#Push into flex table for usable format
IRR_IR_table_flex <- IRR_IR_table |>
  flextable() |>
  set_caption(caption = as_paragraph(
    as_chunk(
      "Risk Bins Across Time Horizons Incidence Rates",
      props = fp_text_default(bold = TRUE)
    )
  )) |>
```

```
  add_footer_lines(
    "Table x: Incidence rates (IR) ± Standard Error (se); Incidence rate ratios (IRR) [95% Confidence Interva
l; Reference group[quantile 1] compared to 2, 3, 4 quantile within each time horizon."
  )

#Save flex table
IRR_IR_table_flex |> save_as_docx(path = "IRR_IR_table_flex.docx")
```

Plot Survival Curves

```
censored_test_data <-
  test_data_with_risks |>
  mutate(
    eventtime = ifelse(all_encounter_date > 365,
                      365, all_encounter_date),
    status = ifelse(all_encounter_date > 365,
                   0, all_msk),
    surv_censored = Surv(eventtime, status)
  )

risk_group_km <- survfit(surv_censored ~ risk_group_180,
                        data = censored_test_data)



#Get Median Survival Probability
risk_group_km <- survfit(surv_censored ~ risk_group_180,
                        data = censored_test_data)

risk_group_km



#extract the specific time horizons
km_plot <- ggsurvplot(
  risk_group_km,
  conf.int = TRUE,
  risk.table = TRUE,
  legend.title = "Risk Group",
  legend.labs = c(1, 2, 3, 4),
  legend = "right",
  tables.y.text.col = FALSE,
  xlim = c(0, 365),
  break.x.by = 1

)



#extract ggplot from survplot
km_plot_1 <- km_plot$plot
km_plot_1 <- km_plot_1 + scale_x_continuous(breaks = c(0, 30, 90, 180, 365))

km_plot_1

#extract table from survplot
tab <- km_plot$table
tab$layers = NULL # clear labels
tab <- tab +
  geom_text(aes(
    x = time,
    y = rev(strata),
    label = llabels
  ), data = tab$data[tab$data$time %in% c(0, 30, 90, 180, 365), ]) +
  scale_x_continuous(breaks = c(0, 30, 90, 180, 365))
```

```r
# Add plots back
km_plot$plot <- km_plot_1
km_plot$table <- tab

km_plot

#Save the KM Curve with Risk Table (comment out to knit into word)
# jpeg(
#   "KM_Curve.jpeg",
#   res = 300,
#   width = 7,
#   height = 7,
#   pointsize = 1 / 300,
#   units = 'in'
# )
# km_plot
# dev.off()
```

Descriptive Statistics

Start by looking at summary statistics of each risk group.

Factors

For factors, calculate proportions of each level.

```r
summarize_factor <- function(data, risk_group_str, feature) {
  data |>
    group_by(!! sym(risk_group_str), !! sym(feature)) |>
    summarize(n = n(), .groups = "drop_last") |>
    mutate(freq = n / sum(n),
           se = sqrt(freq * (1 - freq) / sum(n))) |>
    ungroup() |>
    mutate(quantile = as.numeric(!!sym(risk_group_str)),
           time = parse_number(risk_group_str),
           risk_bin = !! sym(risk_group_str)) |>
    select(- !! sym(risk_group_str)) |>
    relocate(quantile)
}

factor_summary <-
  map(factor_cols,
    \(x) map_df(
      risk_groups,
      \(y) summarize_factor(test_data_with_risks, y, x)
    ))
print(factor_summary)
```

It is easier to visualize the differences in plots.

```r
plot_factor_summary <- function(factor_summary) {
  feature_name <- colnames(factor_summary)[2]
  dodge_width <- 0.9

  factor_summary |>
    filter(time == 180) |>
    ggplot(aes(fill=!! sym(feature_name), y=freq, x=quantile)) +
      geom_bar(position=position_dodge(dodge_width), stat="identity") +
      geom_errorbar(aes(ymin = freq - 1.96*se,
                        ymax = freq + 1.96*se),
                    position = position_dodge(dodge_width),
                    width = 0.5) +
```

```
      geom_text(aes(label = paste0(round(freq*100, 0),"%"),
                    y = freq / 2),
                position = position_dodge(dodge_width),
                size = 4) +
      xlab("Quantile") +
      ylab("Proportion") +
      theme_bw()
}

factor_plots <- map(factor_summary, plot_factor_summary)
factor_plots
```

Numeric

For numeric features, calculate min, max, median, mean, and standard deviation.

```
summarize_numeric <- function(data, risk_group_str, feature) {
  data |>
    group_by(!! sym(risk_group_str)) |>
    summarize(median = median(!! sym(feature)),
              mean = mean(!! sym(feature)),
              sd = sd(!! sym(feature)),
              min = min(!! sym(feature)),
              max = max(!! sym(feature)),
              n = n(),
              .groups = "drop_last") |>
    ungroup() |>
    mutate(quantile = as.numeric(!!sym(risk_group_str)),
           time = parse_number(risk_group_str),
           risk_bin = !! sym(risk_group_str),
           sem = sd / sqrt(n),
           feature_name = feature) |>
    select(- !! sym(risk_group_str)) |>
    relocate(quantile) |>
    relocate(feature_name, .after = quantile) |>
    relocate(sem, .after = sd)
}

map(numeric_covariates,
    \(x) map_df(
      risk_groups,
      \(y) summarize_numeric(test_data_with_risks, y, x)
    ))
```

Post-Hoc Comparisons

First find the proportions of categorical variables in each risk group and compare with a chi-squared test.

```
evaluate_factors_posthoc <- function(data, feature, risk_group) {
  # p-value for all levels
  p_value<- fisher.test(data[[risk_group]], data[[feature]],
                        simulate.p.value = TRUE, B = 100000)$p.value

  # p-value for comparison to risk group 1
  p <- map_dbl(
    c(2, 3, 4),
    \(x) data |>
      mutate(quantile = as.numeric(!!sym(risk_group))) |>
      filter(quantile %in% c(1, x)) |>
      select(!!sym(risk_group), !!sym(feature)) |>
      droplevels() |>
      table() |>
      fisher.test(simulate.p.value = TRUE, B = 100000) %>%
      .$p.value
```

```
  )

  p_df <-
    tibble(value = p, name = c("p_2", "p_3", "p_4")) |>
    pivot_wider()

  data |>
    group_by(!!sym(risk_group),!!sym(feature)) |>
    summarize(n = n(), .groups = "drop_last") |>
    mutate(freq = n / sum(n) * 100,
           freq = paste0(format(round(freq, 1), nsmall = 1), "%")) |>
    ungroup() |>
    mutate(quantile = as.numeric(!!sym(risk_group))) |>
    select(-c(!!sym(risk_group), n)) |>
    relocate(quantile) |>
    pivot_wider(names_from = "quantile", values_from = "freq") |>
    mutate(Feature = feature,
           p_value = p_value) |>
    relocate(Feature) |>
    rename(Levels = feature) |>
    bind_cols(p_df) |>
    rename("One" = `1`,
           "Two" = `2`,
           "Three" = `3`,
           "Four" = `4`)
}

post_hoc_factors <-
  map_df(
    factor_cols,
    \(x) evaluate_factors_posthoc(test_data_with_risks, x, "risk_group_180")
  )

print(post_hoc_factors)
```

Next calculate the means of numeric features and compare with univariate ANOVA.

```
#Mean and ANOVA test
evaluate_numeric_posthoc <- function(data, feature, risk_group) {
  p_value <-
    aov(formula(paste0(feature, "~", risk_group)), data) |>
    tidy() |>
    pull(p.value) |>
    first()

  baseline <-
    data |>
    mutate(quantile = as.numeric(!! sym(risk_group))) |>
    filter(quantile == 1) |>
    pull(!! sym(feature))

  data |>
    mutate(quantile = as.numeric(!! sym(risk_group))) |>
    group_by(quantile) |>
    summarize(mean = mean(!! sym(feature)),
              sd = sd(!! sym(feature)),
              Mean = paste0(format(round(mean, 1), nsmall = 1),
                            "±",
                            format(round(sd, 1), nsmall = 1)),
              p = t.test(!! sym(feature), baseline)$p.value,
              .groups = "drop_last") |>
    ungroup() |>
    select(- c(mean, sd)) |>
    relocate(quantile) |>
```

```r
    pivot_wider(names_from = "quantile", values_from = c("Mean", "p")) |>
    mutate(Feature = feature,
           p_value = p_value) |>
    relocate(Feature) |>
    rename("One" = "Mean_1",
           "Two" = "Mean_2",
           "Three" = "Mean_3",
           "Four" = "Mean_4") |>
    select(-"p_1")
}

post_hoc_numeric <-
  map_df(
    numeric_covariates,
    \(x) evaluate_numeric_posthoc(test_data_with_risks, x, "risk_group_180")
  )
print(post_hoc_numeric)
```

Now combine results and correct p-values.

```r
# combine numeric and factor
post_hoc_summary <-
  bind_rows(post_hoc_factors, post_hoc_numeric) |>
  mutate(Feature = as.factor(Feature))

# correct all p-values together
post_hoc_summary <-
  post_hoc_summary |>
  pivot_longer(cols = starts_with("p_")) |>
  group_by(Feature, name) |>
  mutate(value = unique(value)) |>
  ungroup() |>
  mutate(value = p.adjust(value, method = "holm")) |>
  pivot_wider()

# rename p-value
post_hoc_summary <-
  post_hoc_summary |>
  mutate(`p value` = p_value) |>
  select(-p_value)

# indicate significance
post_hoc_summary <-
  post_hoc_summary |>
  mutate(significance = map_chr(`p value`, decorate_significance),
         Two = paste0(Two, map_chr(p_2, decorate_significance)),
         Three = paste0(Three, map_chr(p_3, decorate_significance)),
         Four = paste0(Four, map_chr(p_4, decorate_significance)))

print(post_hoc_summary)

format_scientific <- function(x) {
  formatC(x, format = "e", digits = 2)
}

format_scientific <- function(x) {
  formatC(x, digits = 4)
}



#Push into flex table for usable format
```

```
post_hoc_table <- post_hoc_summary |>
  flextable() |>
  set_caption(
    caption = as_paragraph(
              as_chunk("Risk Bin Descriptive Statistic", props = fp_text_default( bold = TRUE)))) %>%
  add_footer_lines("Table x: Descriptives statistics for each risk bin; Catagorical predictors are proportion
s (%) across the grouping predictor; Numeric predictors are means for each predictor") |>
  set_formatter(`p value` = format_scientific,
                p_2 = format_scientific,
                p_3 = format_scientific,
                p_4 = format_scientific)

print(post_hoc_table)

#Save flex table
post_hoc_table |>
  save_as_docx(path = "post_hoc_table_flex.docx")
```

Explanations

Global Explanations

Cox Coefficients

```
cox_summary <- tidy(cox)

#Clean factor levels
cox_summary <- cox_summary |>
  tidyr::separate(
    term,
    c("a", "b"),
    "(?<=[a-z])(?=[1,2,3,4]|[A-Z])",
    extra = "merge",
    remove = FALSE
  )      |>
  mutate(a = str_replace(a, "_", " ")) |>
  unite(Term, a, b, remove = FALSE, sep = " : ") |>
  mutate(Term = str_replace(Term, " : NA", "")) |>
  mutate(Term = str_replace(Term, "Movement Pain : 1", "Movement Pain : One")) |>
  mutate(Term = str_replace(Term, "Movement Pain : 2", "Movement Pain : Two")) |>
  mutate(Term = str_replace(Term, "Movement Pain : 3", "Movement Pain : Three")) |>
  mutate(Term = str_replace(Term, " : 1", " : Yes")) |>
  select(-c(term, a, b))|>
  mutate(across(Term, str_replace, 'Prior Mski', 'Prior MSKI'))



cox_summary <- cox_summary |>
  mutate(
    Term = fct_reorder(Term, estimate),
    is_significant = as.factor(ifelse(p.value < 0.05, "Yes", "No")),
    rank = dense_rank(desc(abs(estimate)))
  )


##Coefficients

#Get plot for coefficients
cox_figure <- cox_summary |>
  ggplot(aes(x = estimate,
             y = Term,
             color = is_significant)) +
  geom_point() +
```

```
  geom_errorbar(aes(
    xmin = estimate - 1.96 * std.error,
    xmax = estimate + 1.96 * std.error
  ),
  width = 0) +
  geom_vline(xintercept = 0, linetype = "dashed") +
  scale_color_manual(values = c("black", "red")) +
  guides(color = "none") +
  ggtitle("Cox Coefficient Results") +
  xlab("Coefficient") +
  ylab("Term") +
  theme_bw()


cox_figure

ggsave(cox_figure, file = "cox_figure.jpeg", dpi = 300)


##Hazards

#Calculate Hazard Ratio
cox_summary_2 <- cox_summary |>
  mutate(
    hazard_ratio = exp(estimate),
    lower_CI = exp(estimate - 1.96 * std.error),
    upper_CI = exp(estimate + 1.96 * std.error),
    term = fct_reorder(Term, hazard_ratio),
    is_significant = as.factor(ifelse(p.value < 0.05, "Yes", "No")),
    rank = dense_rank(desc(abs(hazard_ratio)))

  )


#Cox Hazards

cox_figure_2 <- cox_summary_2 |>
  ggplot(aes(x = hazard_ratio,
             y = Term,
             color = is_significant)) +
  geom_point() +
  geom_errorbar(aes(
    xmin = exp(estimate - 1.96 * std.error),
    xmax = exp(estimate + 1.96 * std.error)
  ),
  width = 0) +
  geom_vline(xintercept = 1, linetype = "dashed") +
  scale_color_manual(values = c("black", "red")) +
  guides(color = "none") +
  ggtitle("Cox Hazard Ratios") +
  xlab("Hazard Ratio") +
  ylab("Variable : Level") +
  theme_bw() +
  scale_x_continuous(breaks = seq(-2,8, by = 1) )


cox_figure_2

ggsave(cox_figure_2, file = "cox_figure_2.jpeg", dpi = 300)
```

Plotting the Cox coefficients helps us identify which features were most important in predictions. We can see that the three features that were simulated to have effects all appear as significant, with Gender and BMI having the greatest impacts. It is worth noting that even the statistically insignificant

terms have fairly substantial coefficient values. These will have measurable impacts on predictions from the model.

Random Survival Forest

```
rsf_summary <- rsf |>
  importance() |>
  sort(decreasing=TRUE) |>
  stack() |>
  as_tibble() |>
  rename(var = ind) |>
  mutate(var = fct_reorder(var, values),
         rank = dense_rank(desc(values)))


rsf_VIP_figure<-rsf_summary |>
  ggplot(aes(x=values,
             y=var)) +
  geom_bar(stat="identity") +
  xlab("Importance") +
  ylab("") +
  theme_bw()

ggsave(rsf_VIP_figure, file = "rsf_VIP_figure.jpeg", dpi = 300 )
```

C-Tree

We can also visualize the ctree predictions.

```
custom_node_plot <- function (ctreeobj, ylines = 2, id = TRUE, ...)
{
    survobj <- response(ctreeobj)[[1]]
    if (!("Surv" %in% class(survobj)))
        stop(sQuote("ctreeobj"), " is not a survival tree")
    survdata <- tibble(surv = survobj)
    rval <- function(node) {
        weights <- node$weights
        km <- survfit(surv ~ 1, data=survdata[weights>0, ])
        a <- party:::dostep(km$time, km$surv)
        median_time <- summary(km)$table[["median"]]
        yscale <- c(0, 1)
        xscale <- c(0, max(survobj[, 1]))

        top_vp <- viewport(
          layout = grid.layout(
            nrow=3, ncol=3,
            widths=unit(c(ylines, 1, 1), c("lines", "null", "lines")),
            heights=unit(c(1, 1, 1), c("lines", "lines", "null"))
          ),
          width = unit(1, "npc"),
          height = unit(1, "npc") - unit(2, "lines"),
          name = paste0("node_surv", node$nodeID)
        )
        pushViewport(top_vp)
        grid.rect(gp = gpar(fill = "white", col = 0))

        top <- viewport(layout.pos.col = 2, layout.pos.row = 1)
        pushViewport(top)
        title <- paste(ifelse(id, paste("Node", node$nodeID,
            "(n = "), "n = "), sum(node$weights), ifelse(id,
            ")", ""), sep = "")
        grid.text(title)
        popViewport()
```

```
        subtop <- viewport(layout.pos.col = 2, layout.pos.row = 2)
        pushViewport(subtop)
        subtitle <- paste0("Median Time: ", round(median_time))
        grid.text(subtitle)
        popViewport()

        plot <- viewport(
          layout.pos.col=2, layout.pos.row=3,
          xscale=xscale, yscale=yscale,
          name="plot"
        )
        pushViewport(plot)
        grid.lines(a$x/max(survobj[, 1]), a$y)
        grid.xaxis()
        grid.yaxis()
        grid.rect(gp = gpar(fill = "transparent"))

        upViewport(2)
    }
    return(rval)
}
class(custom_node_plot) <- "grapcon_generator"

#Plot
plot(ctree$ctree, terminal_panel=custom_node_plot)

#Get text results
ctree$ctree



#Adjust the width and height to match the saved file
jpeg("ctree.jpeg", width = 6500, height = 3500, res = 300)
plot(ctree$ctree, terminal_panel=custom_node_plot)
dev.off()

ctree$ctree
```

Get Basic Descriptive Statistics

Incidence Rate

Add grouping factor in #group_by() in the function to add a grouping factor, adjust the function implementation if using #group_by()

```
#Incidence Rate Function
Irate_no_loop<-function(data, time, msk) {
  results <-
  data |>
  #group_by( {{ group }} ) %>%
  summarise(Total_time =  (sum({{time}})/362.25) ,
              Total_msk  =  (sum({{msk}}))) |>
  mutate(cases_per_1000_person_years = ((Total_msk/Total_time)*1000))
  print(data.frame(results))

}

#Function implementation
Irate_no_loop(test_data_with_risks, all_encounter_date, all_msk)
Irate_no_loop(train_data, all_encounter_date, all_msk)
Irate_no_loop(data, all_encounter_date, all_msk)
```

Descriptives

Add grouping factor in #group_by()

```
data |>
  #group_by(Gender)|>
  summarize(
            avg_mass = mean(Mass),
            sd_mass = sd(Mass),
            avg_height = mean(Height),
            sd_height = sd(Height),
            avg_age = mean(Age),
            sd_age = sd(Age),
            n = n())|>
   mutate(freq = n / sum(n))
```
