# Supplementary figures and images for: A machine learning framework to classify musculoskeletal injury risk groups in military service members
Source: Front Artif Intell. 2024 Jun 19;7:1420210. doi: 10.3389/frai.2024.1420210 (PMC11325721; doi:10.3389/frai.2024.1420210)

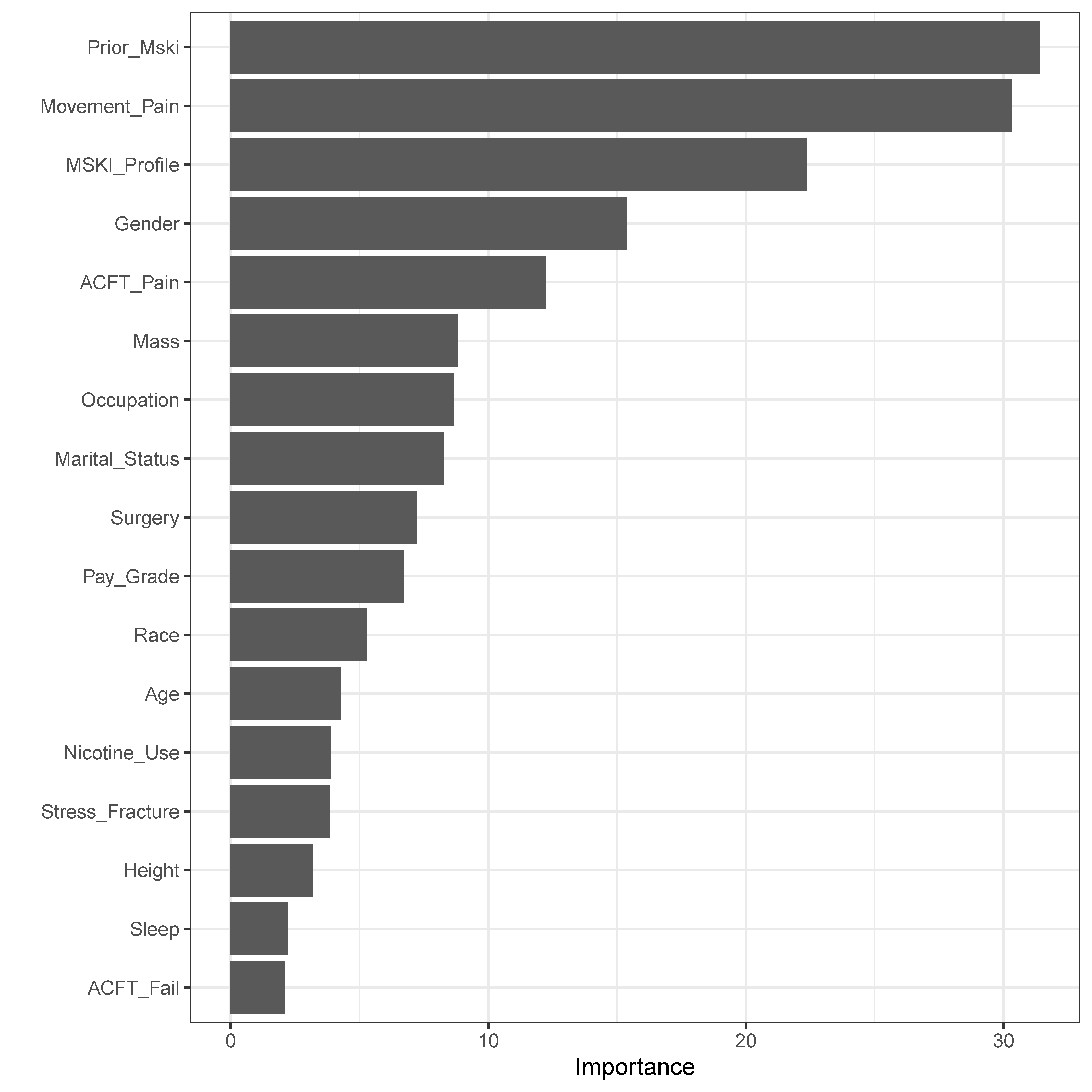

Supplement: Supplementary Figure 1 — Random forest (RF) variable importance. [file Image_1.TIF]
